# Identification of Distinct Tumor Subpopulations in Lung Adenocarcinoma via Single-Cell RNA-seq

**DOI:** 10.1371/journal.pone.0135817

**Published:** 2015-08-25

**Authors:** Jae-Woong Min, Woo Jin Kim, Jeong A. Han, Yu-Jin Jung, Kyu-Tae Kim, Woong-Yang Park, Hae-Ock Lee, Sun Shim Choi

**Affiliations:** 1 Department of Medical Biotechnology, College of Biomedical Science, and Institute of Bioscience & Biotechnology, Kangwon National University, Chuncheon, 200–701, Korea; 2 School of Medicine, Kangwon National University, Chuncheon, 200–701, Korea; 3 Department of Biological Sciences, Kangwon National University, Chuncheon, 200–701, Korea; 4 Samsung Genome Institute, Samsung Medical Center, Seoul, Korea; 5 Department of Molecular Cell Biology, Sungkyunkwan University, Seoul, Korea; University of Navarra, SPAIN

## Abstract

Single-cell sequencing, which is used to detect clinically important tumor subpopulations, is necessary for understanding tumor heterogeneity. Here, we analyzed transcriptomic data obtained from 34 single cells from human lung adenocarcinoma (LADC) patient-derived xenografts (PDXs). To focus on the intrinsic transcriptomic signatures of these tumors, we filtered out genes that displayed extensive expression changes following xenografting and cell culture. Then, we performed clustering analysis using co-regulated gene modules rather than individual genes to minimize read drop-out errors associated with single-cell sequencing. This combined approach revealed two distinct intra-tumoral subgroups that were primarily distinguished by the gene module G64. The G64 module was predominantly composed of cell-cycle genes. E2F1 was found to be the transcription factor that most likely mediates the expression of the G64 module in single LADC cells. Interestingly, the G64 module also indicated inter-tumoral heterogeneity based on its association with patient survival and other clinical variables such as smoking status and tumor stage. Taken together, these results demonstrate the feasibility of single-cell RNA sequencing and the strength of our analytical pipeline for the identification of tumor subpopulations.

## Introduction

Tumors are not identical between patients at the molecular or morphological/pathological level. This observation, often referred to as inter-tumoral heterogeneity, forms the basis of targeted cancer medicine [1–3]. Further tumor heterogeneity is present within a single patient; this phenomenon is defined as ‘intra-tumoral heterogeneity’ [4–10], and its clinical importance is increasingly being recognized [11–14]. Distinct tumor subpopulations, often harboring different genetic mutations, may have varying sensitivities to targeted treatments [15–19], and drug-resistant subclones may cause treatment failure.

The extent of tumor heterogeneity has been studied at a genomic level by inferring subclones from deep sequencing [20–22]. Several groups have used multi-regional sequencing approaches to study intra-tumoral heterogeneity in breast cancer [23] and glioblastoma [7]. More recently, it was demonstrated that single-cell sequencing is necessary to identify the actual number of subclones in a tumor, and this method has revealed the number of subclones and evolutionary patterns in many cancer types [24–26]. Although various approaches can be adopted for the study of DNA-level heterogeneity, gene expression-level heterogeneity in mixed populations is difficult to assess by bulk sequencing. Indeed, Patel et al. [27] performed single-cell RNA sequencing (RNA-seq) analysis on 430 single glioblastoma cells from five patients and demonstrated cellular heterogeneity in transcriptional programs involved in oncogenic signaling, proliferation, and hypoxia. The results of their study suggest that single-cell transcriptomic analysis may reveal clinically important subpopulations within heterogeneous tumor cell populations.

In the present study, we assessed the transcriptional characteristics of 34 patient-derived xenograft (PDX) cells originating from an LADC tumor region at a single-cell resolution [28]. Characterization of the PDX has become an important issue, as this model is increasingly used as a drug screening platform [29]. To focus on the intrinsic tumor transcriptome, we first filtered out differential gene expression associated with xenografting and cell culture. Then, we selected gene modules that were highly and correlatively expressed across 34 single LADC cells. Individual cells were then clustered according to the gene expression modules to determine the subgroup profiles among the 34 single cells. One gene module, termed G64, divided the single cells into 2 distinct subpopulations and separated 488 LADC patients into subgroups based on samples from the Cancer Genome Atlas (TCGA). The G64 up-regulated group was associated with poor prognosis and other clinical variables such as smoking and advanced tumor stage.

## Materials and Methods

This study was performed in accordance with the principles of the Declaration of Helsinki and was approved by The Samsung Medical Center (Seoul, Korea) Institutional Review Board (IRB) (No. 2010-04-004).

### Sample Description

The patient sample was originally described by Kim et al. [28]. Briefly, the tumor specimen was a 37-mm irregular primary lung lesion from a 60-year-old male and was determined to be a poorly differentiated lung adenocarcinoma harboring wild-type EGFR and G12D mutant KRAS. A portion of the primary tumor tissue was transplanted into the subrenal space of a humanized immunocompromised female NOG mouse, and the transplant was expanded to obtain a xenograft tumor. The xenograft tumor was dissociated into a single-cell suspension and was cultured for fewer than 3 in vitro passages before single-cell capture and RNA sequencing. The variant allele frequency of the KRAS G12D mutation was 0.04 for the primary tumor tissue but was >0.8 for the xenograft tissue and the cultured cells. Single-cell DNA and RNA analyses revealed that all cells harbored the KRAS G12D mutation at variable DNA copy numbers and that 27 of the 34 individual cells expressed a detectable level of G12D KRAS transcripts.

### Sources of RNA-seq Data and Processing Procedures

We used single-cell and bulk RNA-seq data generated by Kim et al. [28], which have been deposited in the Sequence Read Archive (SRA, NCBI) using the Gene Expression Omnibus (GEO) (GSE69405). The RNA-seq data for the primary matched normal (pN) and mouse xenograft (xenoT) samples can be accessed in the GEO by GSE70968. According to Kim et al. [28], single-cell capture and cDNA amplification were performed using a SMARTer kit (Clontech, Mountain View, CA) on a 17–25 μm microfluidic chip (Fluidigm, San Francisco, CA). Sequencing libraries were generated using a Nextera XT DNA Sample Prep Kit (Illumina, San Diego, CA) for single cells or a TruSeq RNA Sample Preparation v2 kit (Illumina) for bulk RNA samples, and sequencing reads were generated using HiSeq2500 sequencing system in the 100-bp paired-end mode using a TruSeq Rapid PE Cluster kit and a TruSeq Rapid SBS kit (Illumina). RNA-seq reads were filtered at Q33 using Trimmomatic-0.30 [30] and were aligned to a human genome reference (hg19) using Tophat2 [31]. Transcripts were estimated as fragments per kilobase of transcript per million mapped reads (FPKM) using Cufflinks [31].

### Excluding Genes Modulated by the Xenografting and Cell Culture Procedures

Single-cell RNA-seq data generated from a PDX cell culture might have noisy expression changes due to the xenografting, cell culture, or both procedures. Therefore, we excluded the differentially expressed genes (DEGs) modulated by the experimental procedures but not by tumorigenesis. The exclusion steps were performed to obtain DEGs (1) between pT and xenoT, (2) between xenoT and bulkT, and (3) between pT and bulkT (S1A Fig). The DEGs were obtained for each comparison based on the log2 fold-change in each FPKM value. Genes that were detected as DEGs (i.e., log2 fold-change > 2) in any of these comparisons were excluded from further analysis (S1B Fig).

### Data Cleaning and Identification of Highly Correlatively Expressed Genes in Module G64

**Fig 1:**
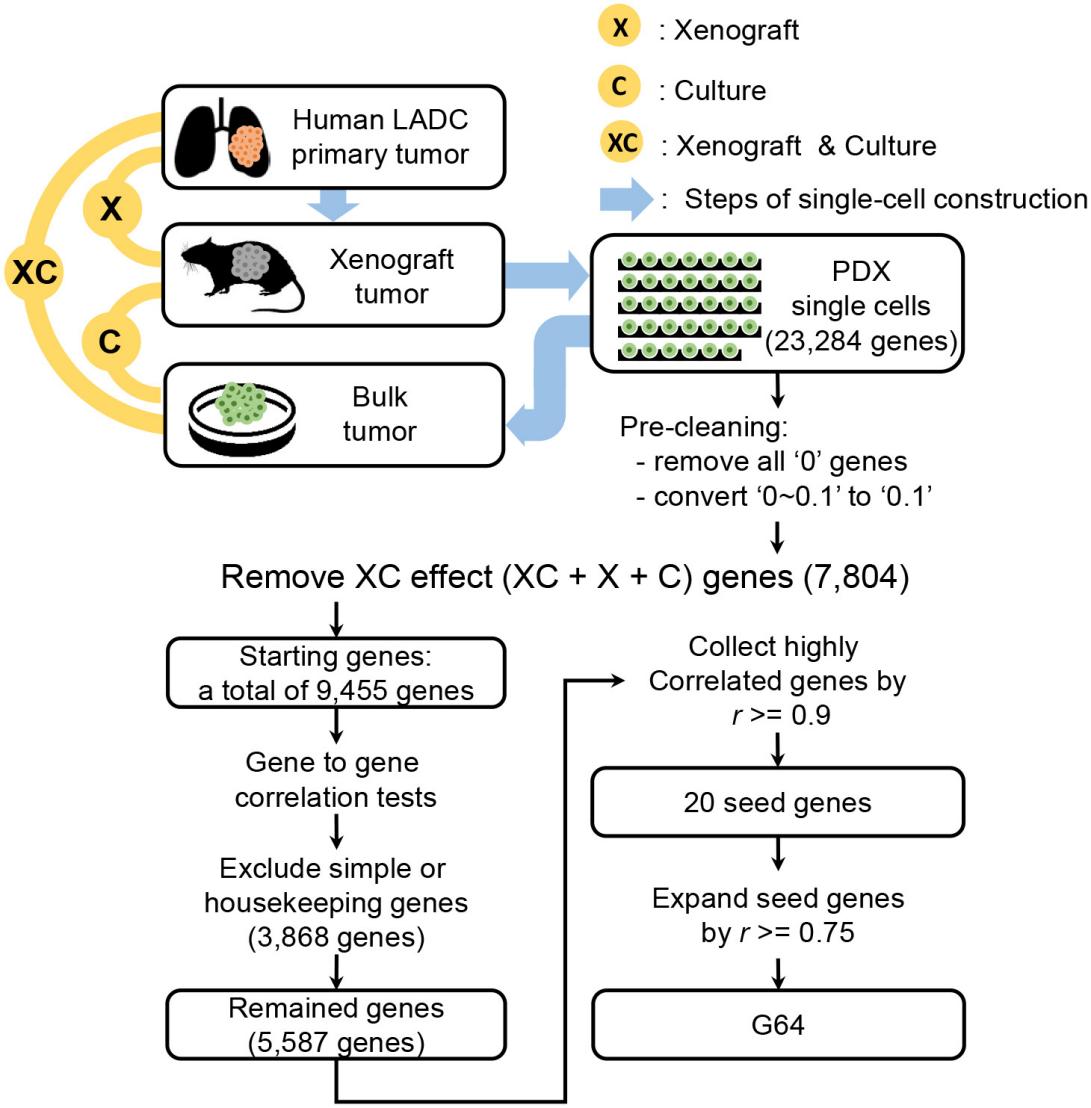
Schematic illustrating the preparation of coordinately co-expressed genes across the 34 single LADC cells. The upper part of the schematic illustrates the sequential procedures of LADC tissue isolation, mouse engraftment, patient-derived xenograft (PDX) cell culture, and single PDX cell preparation for RNA sequencing [28]. The lower part of the schematic explains how the coordinately expressed genes were selected from the 34 single-cell transcriptomes. Please refer to the Materials and Methods section for a detailed description of the procedures.

An overall schematic of this process is presented in Fig 1. Among a total of 23,284 genes in the initial transcriptome, 9,455 genes remained after excluding genes that displayed FPKM values of “0” across the 34 single-cell samples. All FPKM values greater than 0 but less than 0.1 were converted to 0.1 to avoid the infinity problem [32–35] in calculating the expression fold-changes for the remaining 9,455 genes. Then, to determine the correlation of the expression of a given gene with that of another gene in the 34 single cells, gene-to-gene expression correlation between the 34 single cells was estimated via Pearson’s correlation analysis using R (http://www.r-project.org/), resulting in 9,455 x 9,455 comparisons. Next, genes displaying highly correlated expression based on their simple expression patterns (i.e., patterns emerging when few single cells express the given gene) or their ubiquitous expression (i.e., patterns emerging when the expression of the given gene was generally constant across the 34 single cells) were identified for exclusion (S2 Fig), resulting in a total of 5,587 remaining genes. To identify gene modules, the gene groups containing at least five genes displaying a Pearson’s correlation coefficient of (*r*) > = 0.9 were collected, from which a total of 20 genes with highly correlated expression were selected (S1 Table); these genes were considered as ‘seed genes’. The seed genes were then used to expand the number of included genes using a threshold of *r* > = 0.75. Finally, 64 highly correlatively expressed genes across the 34 single cells were identified, and this group of genes was termed “G64” (S1 Table).

### Websites for Downloading the Transcription Factor (TF) Binding Site (TFBS), CycleBase, and TCGA LADC Datasets

The data for the TFBSs that were predicted to be located within the 5 kb upstream regions of the genes in G64 were downloaded from ChIPBase (http://deepbase.sysu.edu.cn/chipbase/protein.php) [36]. Cell-cycle genes and their cell-cycle stages were downloaded from CycleBase (http://www.cyclebase.org/CyclebaseSearch). RNA-seq transcriptome data and patient survival information for 488 human LADC patients were downloaded from the TCGA website (https://tcga-data.nci.nih.gov/tcga/tcgaHome2.jsp).

### Statistical Tests

All statistical tests and analyses were performed using the R studio (http://www.rstudio.com/). The ‘cor.test’ function was used to compute Pearson’s correlation coefficients (http://www.r-project.org/) to determine the correlation of the FPKM values among the different single cells, whereas the ‘hclust’ function was used for hierarchical clustering (https://stat.ethz.ch/R-manual/R-patched/library/stats/html/hclust.html). The ‘factoMineR’ (http://factominer.free.fr), and ‘rgl’ (https://r-forge.r-project.org/projects/rgl/) packages were used for principal component analysis (PCA) and visualization [37]. Combined with the Kaplan-Meier survival curve [38], the ‘survival’ packages were used to estimate the patient survival rates (http://r-forge.r-project.org). The Cox proportional hazard ratio was computed using the ‘coxph’ function in the ‘survival’ packages [38–40]. The other scripting work necessary for several batch jobs was performed using a proprietary *Perl* script.

## Results

### Cleaning of the Transcriptomes of the 34 Single Cells Derived from LADC PDXs

A schematic of our overall analysis procedure is illustrated in Fig 1. The upper part of the schematic shows the preparation of single cells performed by Kim et al. [28]. Briefly, a tissue block from an LADC surgical sample was engrafted into immuno-compromised mice, and the xenograft tumors were cultured for tumor cell expansion, resulting in a total of 34 single cells. Thus, extensive data cleaning strategies were needed to obtain genes expressed purely in the 34 tumor-derived single cells. The first step was to remove genes displayed altered expression during the single-cell preparation procedures, such as xenografting and cell culture, from the set of genes expressed in the single cells. In addition, lack of sequencing depth is a well-recognized technical limitation of the single-cell-based sequencing approach [41–42]. The lower part of the schematic shows how the genes in the G64 module were identified (see details in the Materials and Methods section).

mN, pT, xenoT, and bulkT represent ‘matched normal’, ‘primary tumor’, ‘xenograft tumor’, and ‘bulk tumor grouped by collecting separated single cells’, respectively (Fig 1 and S1A Fig). In fact, when inter-sample correlations of the expression values were compared before removing the genes whose expression was altered by the experimental procedures, the expression correlation between pT and mN was higher than that between pT and xenoT or between pT and bulkT (S3A Fig). Therefore, following the method illustrated in S3B Fig, genes that displayed altered expression during xenografting or cell culture were excluded from further analysis (see details in the Materials and Methods section). A total of 9,455 genes remained after the extensive cleaning procedures, and the expression correlations between pT and bulkT were highly increased, with a Pearson’s correlation coefficient ranging from 0.4 to 0.9 (S4A and S4B Fig) (see details in the Materials and Methods section).

### Identification of Highly Correlated Gene Modules in the 34 Single Cells

Hierarchical clustering analysis was performed on the gene expression data to determine whether the 34 single cells derived from a single tumor region could be subdivided based on their gene expression. It is important to determine which set of genes serves as a good classifier for identifying cell subpopulations. Hierarchical clustering analysis of the expression of 9,455 complete genes was not useful for analyzing the single-cell subpopulations because the expression levels of these genes in each cell were generally similar, as indicated by a Pearson’s correlation coefficient of 0.55–0.89 (S3A Fig); furthermore, there was an excessive amount of noise preventing the generation of clusters (data not shown). However, the cell subpopulations must be determined by groups of genes rather than by a single gene because the up-/down-regulation of a single gene could be noisy among the single cells.

Thus, we decided to obtain groups of genes displaying highly correlated expression across the 34 single cells by estimating these correlations based on genes displaying complex expression patterns among the single cells. This process was selected based on the reasoning that high gene-to-gene correlations could be generated merely by chance when genes are expressed in only one or two single cells or when genes are ubiquitously expressed (S2 Fig; see details in the Materials and Methods sections). The selected genes were further refined based on the reasoning described above, and a total of 5,587 genes were selected for gene-to-gene correlation analysis among the 34 single cells.

**Fig 2:**
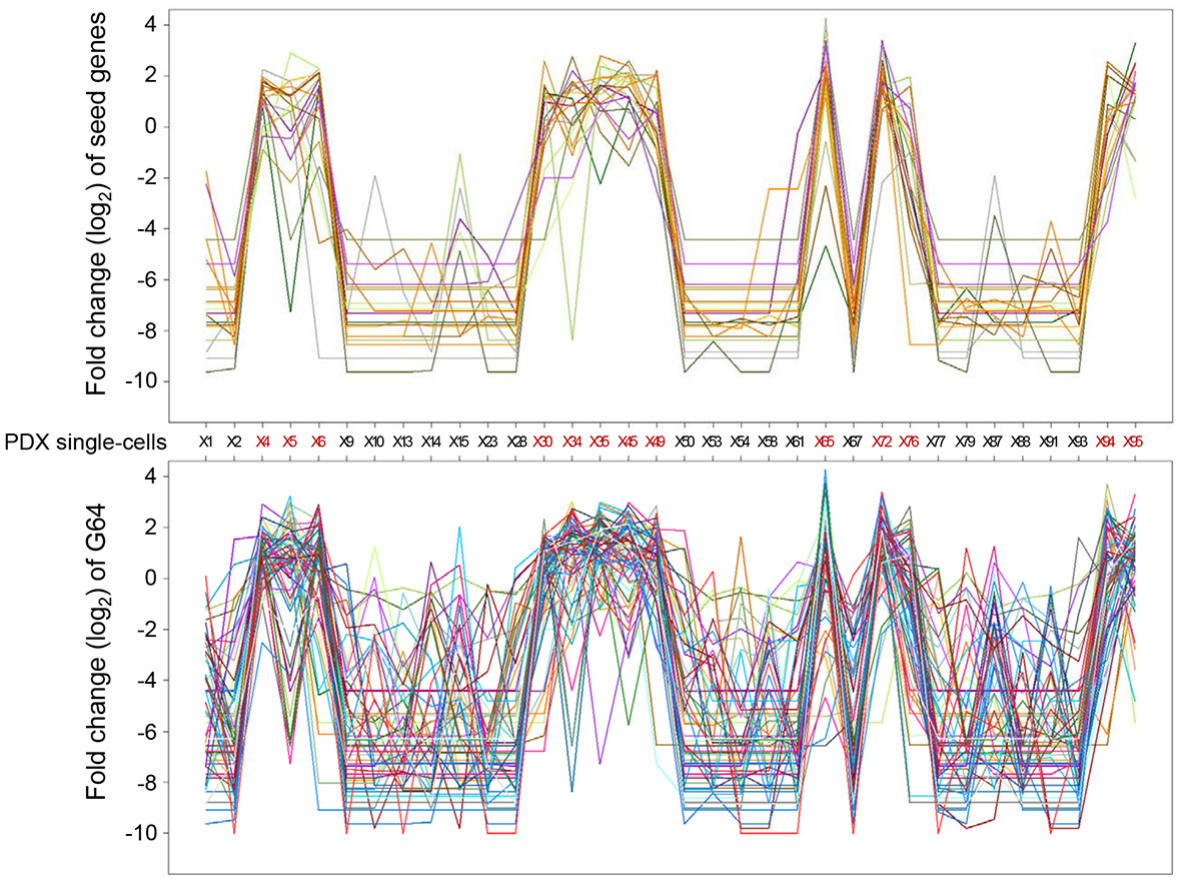
Plotting the expression of seed genes and G64. The log2 fold-change in the expression of a given gene among the single cells compared to the average expression level of the given in the 34 single cells was plotted for seed genes (upper panel) and for the genes in G64 (lower panel). Lines of different color indicate anonymous, distinct genes included in G64.

From the remaining 5,587 x 5,587 gene-to-gene correlations, the gene groups containing at least five genes displaying the highest correlations based on a Pearson’s correlation coefficient of (*r*) > = 0.9 were collected. From this analysis, a total of 20 genes were identified as ‘seed genes’ (in bold in S1 Table). Then, a weaker threshold, r > = 0.75, was applied for the 20 seed genes to expand the gene set, ultimately producing a cluster of 64 genes termed G64 (Fig 2 and S1 Table).

The threshold of 0.9 was arbitrarily selected for collecting the seed genes displaying the most similar expression patterns across the 34 single cells, and a looser threshold of 0.75 was selected to increase the number of genes in the module while maintaining a high correlation among the genes within the module. Note that an excessively loose threshold would expand the number of genes in the module but would reduce the correlation coefficient (S5 Fig).

### Intra-Tumoral Subpopulations and Inter-Patient Groups Distinguished Based on the Expression of G64

**Fig 3:**
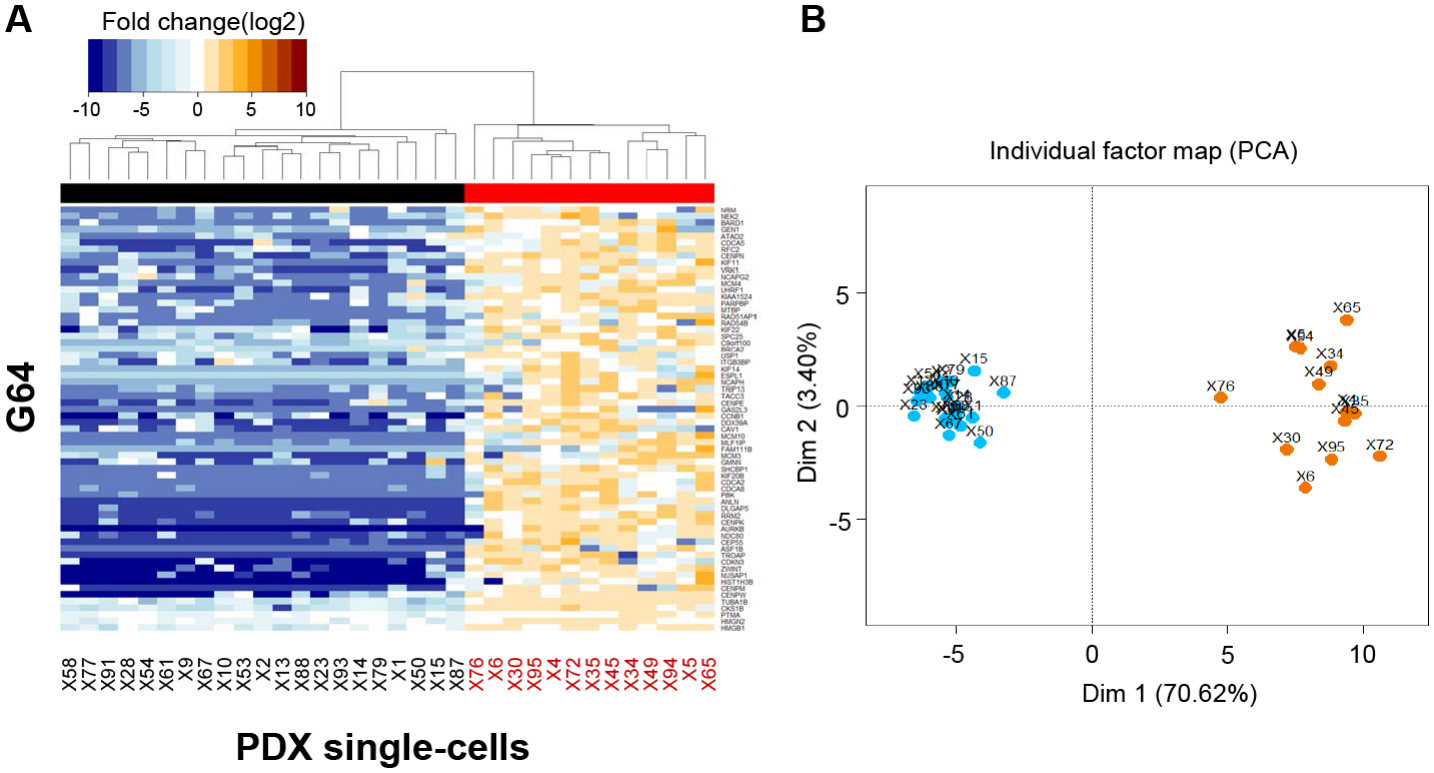
Heat map combined with the hierarchical clustering analysis of G64 expression in the 34 single cells. (A) The 34 single cells were clustered into two subgroups (i.e., single cells displaying G64 down-regulation [left] and single cells displaying G64 up-regulation [right]). See also the dendrogram and black and red flat bars at the top of the heat map. The single cells displaying G64 up-regulation are labeled ‘red’ at the bottom of the heat map to ensure that the single cells exhibiting up-regulated G64 expression could be compared with the single cells shown in the other figures. (B) Principal component analysis of G64 expression in the 34 single cells (see the Materials and Methods section). The same subgroups shown in (A) were clustered. Blue and orange dots represent single cells exhibiting G64 down- and up-regulation, respectively.

Hierarchical clustering was performed on the 34 single cells using G64. The expression fold-change for each gene in each single cell was estimated by dividing the FPKM value in each cell by the average FPKM value of the 34 single cells, followed by log2 transformation. Interestingly, the 34 single cells derived from a single LADC tumor region were divided into two distinct subpopulations according to the expression of G64 (Fig 3A). PCA confirmed this separation (Fig 3B). This result may indicate that single cells derived from even a single tumor are not identical in their gene expression characteristics, and this phenomenon may be related to the different physiological responses of distinct tumor cell subpopulations to drugs or treatments.

**Fig 4:**
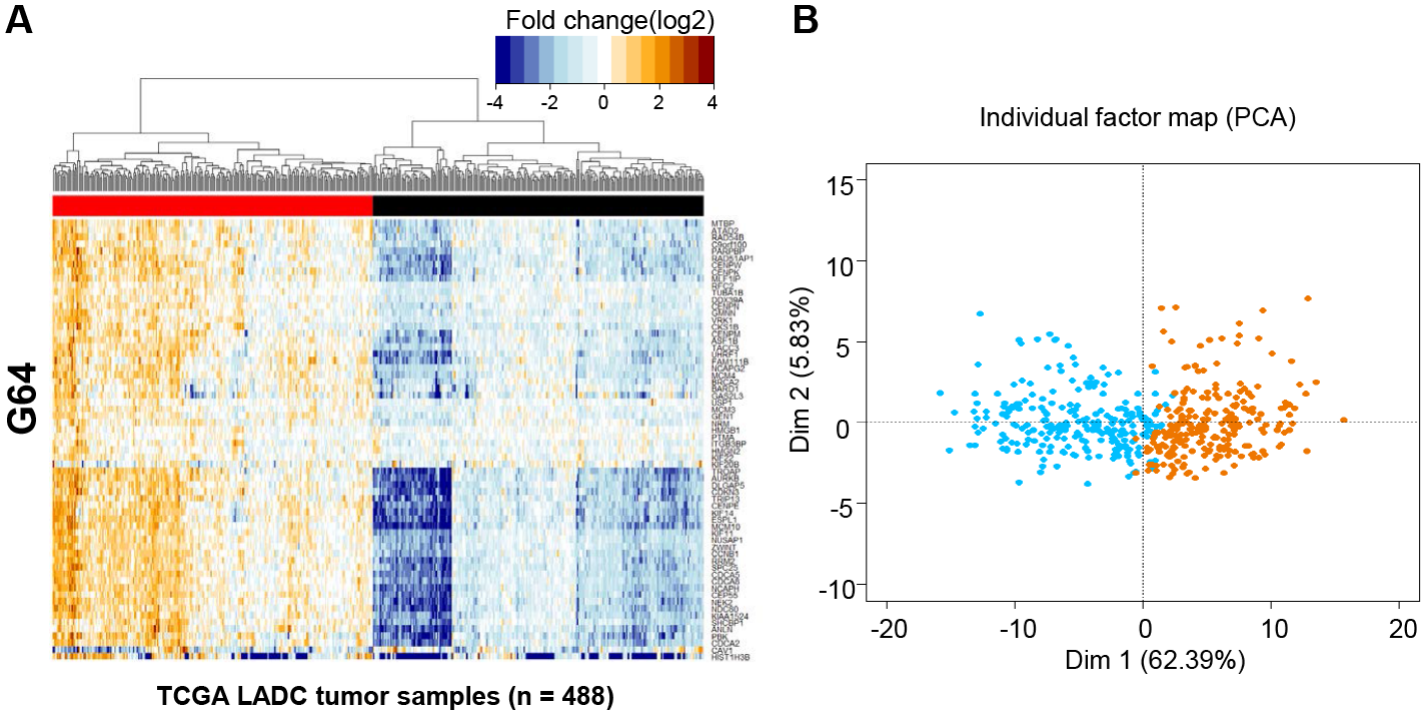
Heat map combined with hierarchical clustering analysis of G64 expression in the 488 LADC samples in TCGA. The 488 LADC tumor samples were divided into two different clusters according to the expression of G64 (i.e., G64 up-regulated tumors (left) and G64 down-regulated tumors [right]) according to the same pattern as shown in Fig 3A. (B) Principal component analysis of G64 expression in the 488 LADC samples in TCGA. The samples were identically separated into two clusters, as represented by blue and orange dots for G64 down- and up-regulation, respectively.

We explored whether the intra-tumoral subgrouping by G64 could be extended to the inter-tumoral level. To this end, transcriptomic data from a total of 488 LADC tumor samples produced by an RNA-seq approach were retrieved from TCGA after 57 paired normal samples were excluded. The fold-change in the expression of each gene in each tumor sample was estimated in the same manner as that for the 34 single cells. Then, hierarchical clustering analysis combined with heat map illustration was performed for the 488 tumor samples. Surprisingly, the G64 genes that distinguished between single cells also clearly differentiated the same subgroups for the LADC samples (Fig 4A). PCA confirmed this differentiation (Fig 4B).

**Fig 5:**
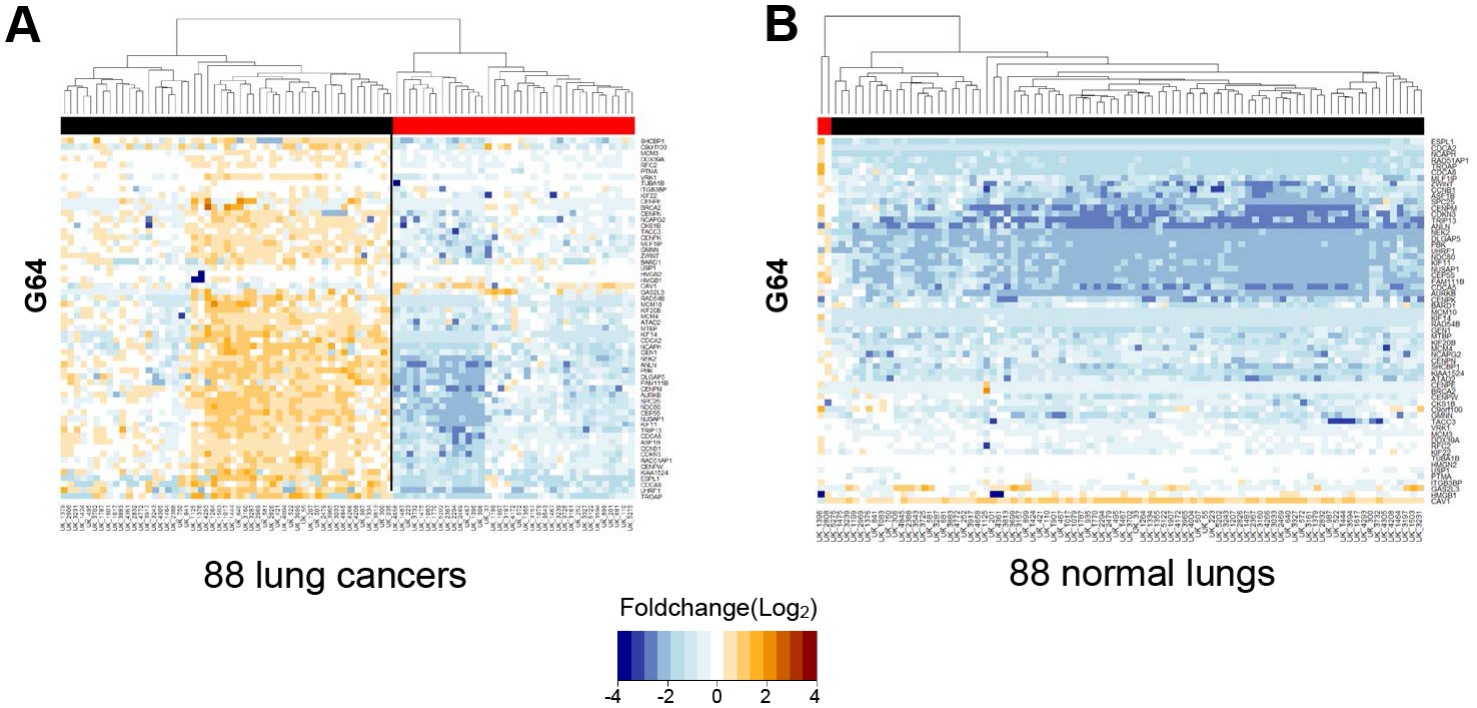
Heat map combined with hierarchical clustering analysis of G64 expression in 88 Korean lung cancer specimens and their paired 88 normal lung samples. The same heat map analysis described in the legend of Fig 3A was performed on RNA-seq data derived from 88 lung cancer specimens (A) and their paired 88 normal lung samples (B), which were obtained from Lee et al. [43].

This two-group separation based on G64 also emerged in the transcriptomic data of 88 Korean lung cancer samples produced using the RNA-seq approach by Lee et al. [43], whereas no such separation appeared in the 88 paired normal lung samples (Fig 5A and 5B). This result indicates that the subgroup classification using G64 may not be limited to the intra-tumoral level but might extend to the inter-tumoral level.

Next, we investigated how the up-/down-regulation of G64 was related to clinical variables of the patients. Univariate analysis was first applied to the 443 LADC cases containing clinical information retrieved from TCGA for the G64 up-regulation cases and the G64 down-regulation cases, and we examined whether each clinical variable was distributed in a significantly different manner between the two groups. As a result, all tested clinical variables, including gender (p = 0.003; odds ratio (O.R.) = 1.731; 95% C.I. = 1.177–2.553), age of initial diagnosis (p = 0.005; O.R. = 1.726; C.I. = 1.162–2.573), stage (p = 0.002; O.R. = 2.014; C.I. = 1.255–3.270), and patient survival (p = 0.000, O.R. = 2.213; C.I. = 1.401–3.533), were significantly associated with the up-/down-regulation of G64 genes based on the univariate chi-squared test (S2 Table). Four different variables related to smoking habits, including tobacco (i.e., smoking or not smoking) (p = 0.043; O.R. = 1.690; C.I. = 0.985–2.935), tobacco year (i.e., years of smoking) (p = 0.052; O.R. = 1.648; C.I. = 0.964–2.831), tobacco reformed year (i.e., years since smoking termination) (p = 0.000; O.R. = 3.106; C.I. = 1.824–5.362), and tobacco pack-years (p = 0.008; O.R. = 1.850; C.I. = 1.141–3.018), were associated with the expression pattern of G64. Interestingly, the heavier smokers, younger patients, and prolonged smokers tended to have higher levels of G64 genes.

Multivariate logistic regression analysis was performed to determine the independent contribution of each clinical variable on the expression of G64 demonstrated, and the results showed that the up-regulation of G64 was strongly associated with poor patient prognosis compared to the G64 down-regulation (i.e., O.R. for survival = 2.101) (Table 1). The O.R. for smoking in particular (we included one of the four variables, ‘tobacco reformed year’ from S2 Table in the multivariate model) displayed the highest tendency toward G64 up-regulation (O.R. = 3.508) (Table 1). Note that only ‘tobacco reformed year’ was used for this multivariate logistic regression analysis among all of the tested smoking variables presented in S2 Table because the difference in its distribution between the G64 up-regulation group and the G64-down regulation group was the greatest (p = 0.000; O.R. = 3.106) (S2 Table).

**Table 1:** Multivariate logistic regression of clinical factors between the G64 up- and down-regulation groups.

	Group		Statistical result		
Factors	Down (228)	Up (234)	*P* ^a^	O.R.^b^	95% C.I. ^c^
Gender	Female: 140 (61.4%), Male: 88 (38.6%)	Female: 112 (47.9%), Male: 122 (52.1%)	0.037	1.789	1.037 ~ 3.085
Age of initial pathologic diagnosis	> = 65: 140 (63.3%), < 65: 81 (36.7%)	> = 65: 111 (50.0%), < 65: 111 (50.0%)	0.322	1.328	0.757 ~ 2.328
Stage	1 ~ 2: 190 (83.7%), 3 ~ 4: 37 (16.3%)	1 ~ 2: 168 (71.8%), 3 ~ 4: 66 (28.2%)	0.013	2.286	1.189 ~ 4.398
Tobacco reformed year	> = 15: 79 (55.2%), < 15: 64 (44.8%)	> = 15: 36 (28.3%), < 15: 91 (71.7%)	0	3.508	1.953 ~ 6.301
Survival status	Survival: 188 (82.5%), Death: 40 (17.5%)	Survival: 159 (67.9%), Death: 75 (32.1%)	0.021	2.101	1.121 ~ 3.938

^a^ *p* value;

^b^odds ratio;

^c^confidence interval

Furthermore, statistical association tests between subpopulations divided according to the expression of G64 and gene mutations such as KRAS, EGFR, and ALK were performed. The results showed that the subpopulations were not significantly associated with these gene mutations (data not shown). Relatedly, most of the single cells that we analyzed in this study harbored the G12D KRAS mutation at variable DNA copy numbers (27/34) [28], and no significant correlation was observed between this mutation and the two single-cell subpopulations.

The co-expression of G64 genes may represent an interesting previously unstudied dimension of cancer, particularly lung adenocarcinoma. Thus, we investigated how G64 genes were differentially expressed in 501 squamous cell lung cancer (LUSC) specimens in TCGA. Interestingly, the 501 LUSC samples also appeared to be separated by G64, although less prominently (S6 Fig). Note that only 21% (n = 107) of the 501 LUSC samples were assigned to the G64 down-regulation group (S6 Fig), whereas approximately 50% of the LADC samples were included in the G64 down-regulation group, indicating that LADC patients are significantly more likely to express G64 at lower levels than LUSC patients (p < 0.0001 for the chi-squared test).

### G64 Genes Were Mainly Classified as Cell-Cycle Genes

To explore the functions of the G64 genes, gene classification analysis was performed using DAVID (http://david.abcc.ncifcrf.gov/), and the G64 genes were significantly assigned to the cell-cycle group (S3 Table).

It may not be surprising to find cell-cycle genes among the set of genes related to tumor cell subpopulations because cell cycle genes in particular have long been known to play key roles in tumor cell proliferation and tumor progression [44–48]. However, it is surprising that cell-cycle genes were coordinately expressed across the 34 single cells. Interestingly, the transcriptomic analysis of 433 single cells derived from glioblastoma (GBM) by Patel et al. [27] also found that cell cycle genes were predominantly and correlatively expressed in the 433 single GBM cells.

**Fig 6:**
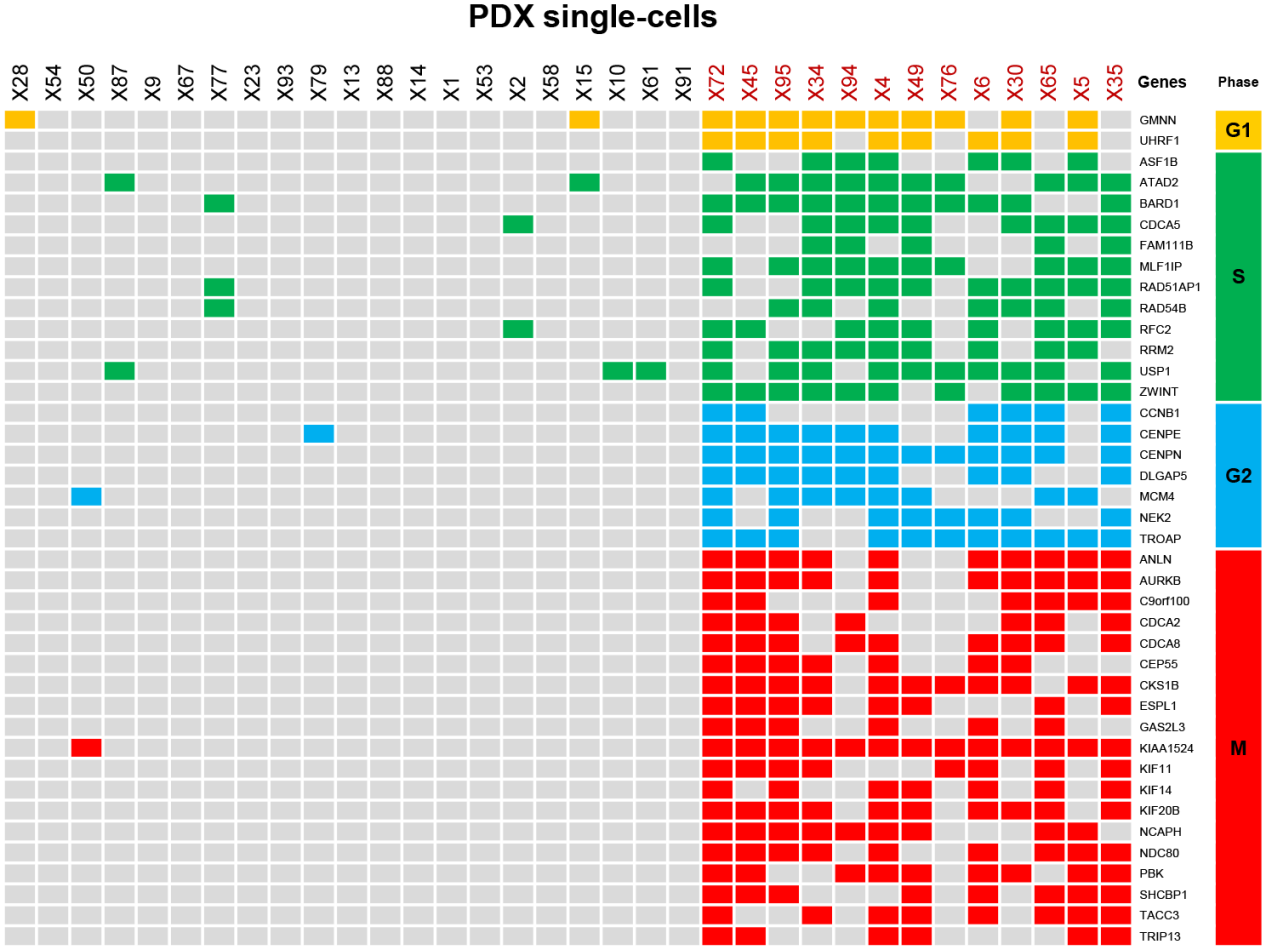
Cell-cycle regulation is a major functional class of G64 genes. A total of 40 genes in the G64 module were mapped to cell-cycle genes in CycleBase (http://www.cyclebase.org/). Each of the 40 cell-cycle genes was accordingly allocated to one of the cell-cycle stages (e.g., G1, S, G2, or M, colored in yellow, green, blue, and red, respectively).

Thus, we examined whether the G64 genes were expressed during a specific cell-cycle stage and whether the two groups of single cells, i.e., the G64 up-regulation group and the G64 down-regulation group, actually represent two different types of cells, i.e., dividing cells and non-dividing cells. Forty G64 genes could be assigned to each corresponding cell-cycle stage based on mapping to the cell-cycle genes in CycleBase (http://www.cyclebase.org/Downloads). In fact, the 40 genes were not restricted to a single cell-cycle stage but rather were found in all different types of cell-cycle stage categories (e.g., the G1, S, G2, and M phases) (Fig 6). Based on this result, all 13 single cells exhibiting G64 up-regulation may actually express different cell-cycle genes acting in different cell-cycle stages, whereas the 21 single cells exhibiting G64 down-regulation express few of these cell-cycle genes. This result indicates that the subpopulations of the 34 single cells stratified by G64 expression are not distinct due to their cell division capabilities. Consistently, as shown in Figs 4 and 5, the differential co-expression of G64 genes was applicable to not only the intra-tumoral level but also the tissue level of the tumors (i.e., different groups of thousands of single cells).

### E2F1, a Known Tumor-Related TF, Likely Serves as the Upstream Regulator of G64

**Fig 7:**
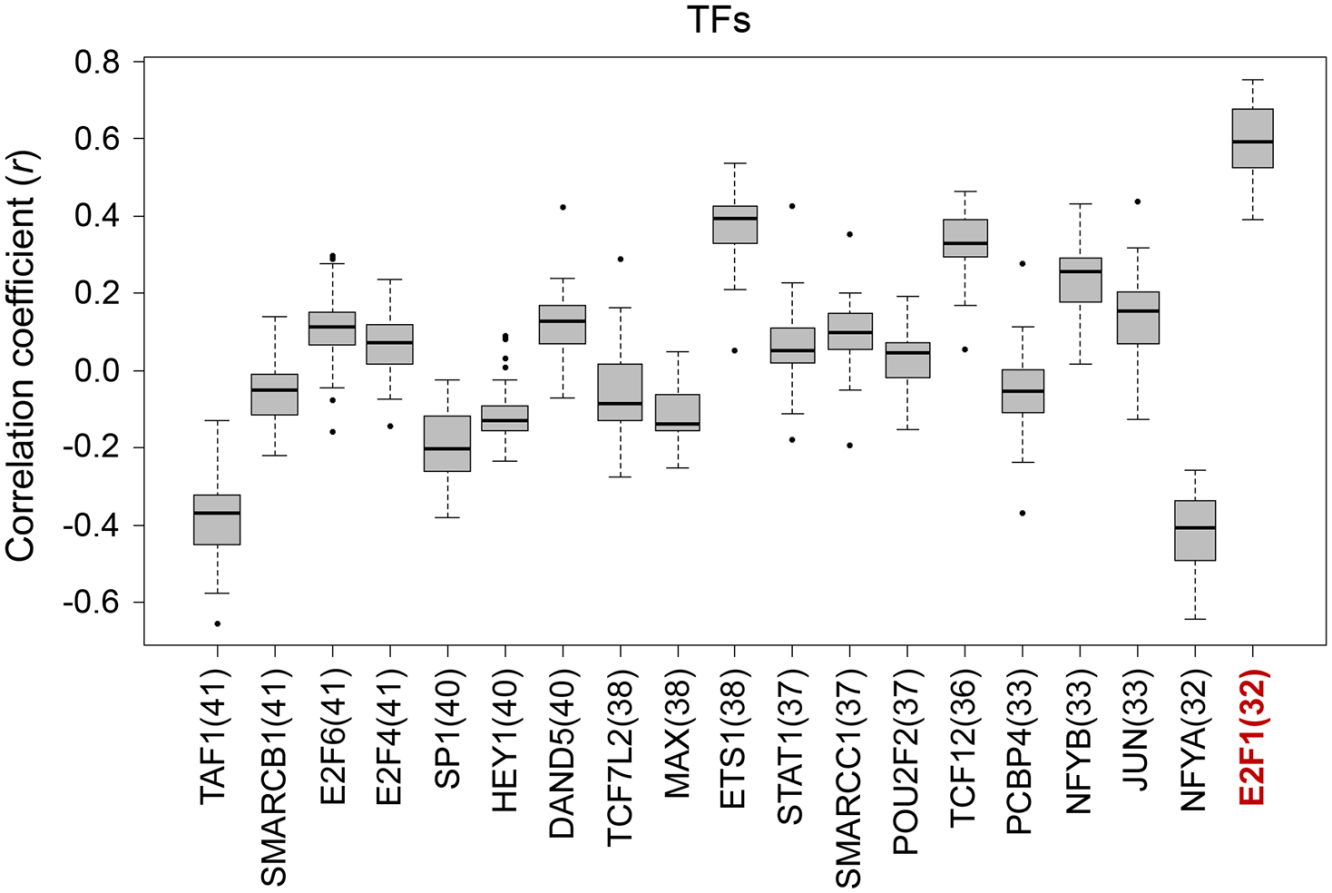
Searching for putative transcription factors that co-regulate G64 expression. (A) A TF with a TFBS located at an upstream region in more than 50% of the G64 genes (i.e., > = 32 of these genes) was considered as a putative TF that commonly regulates G64. Please refer to the Materials and Methods section for details regarding the method used to search for putative TFBSs upstream of G64. Nineteen putative common TFs were found, one of which was E2F1, a well-known gene associated with cancer. Pearson’s correlation coefficients were estimated for the relationship between the expression of each of these TFs and that of G64, and E2F1 displayed the highest correlation coefficient.

Next, we investigated whether there were common TFBSs within the regions 5 kb upstream of G64 genes (see the Materials and Methods section). Nineteen TFs were predicted to be commonly located in the upstream promoter regions of at least thirty-two G64 genes (S7 Fig). We reasoned that if these TFs actually control the expression of G64 genes, then the expression of these TFs would highly correlate with G64 expression. Interestingly, E2F1, a well-known TF that regulates the cell cycle and tumorigenesis [49–52], demonstrated the highest correlation coefficient based on correlation analysis of the expression between the G64 genes and each of the 19 selected TFs (Fig 7). When the same reasoning was applied to examine the 488 LADC patient samples, E2F1 again demonstrated the highest correlation with G64 genes (S8 Fig), strongly suggesting that E2F1 is a plausible candidate upstream regulator responsible for the co-expression of G64.

### Single Cells Exhibiting G64 Up-Regulation Tend to Have a Poor Prognosis

**Fig 8:**
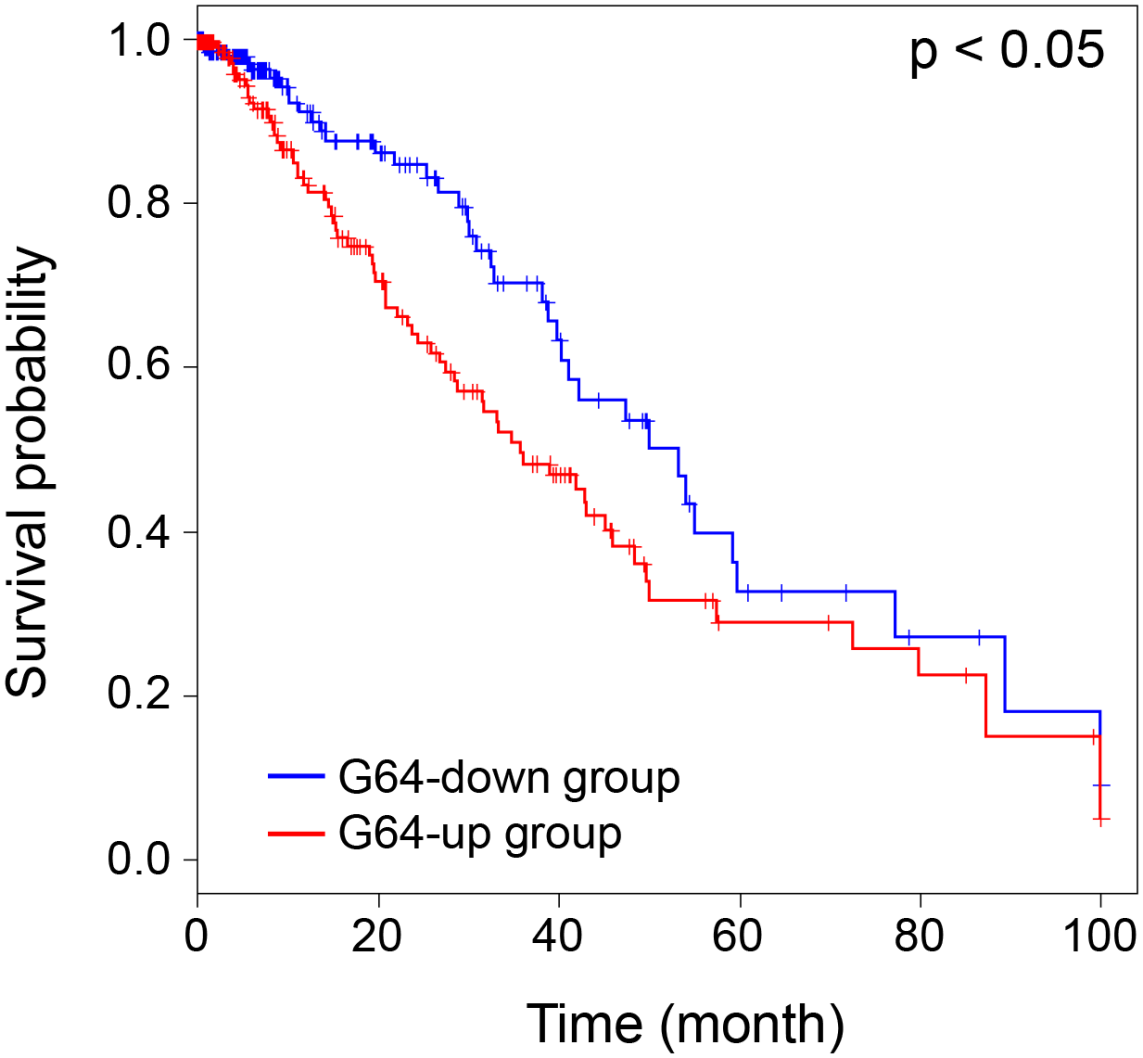
Kaplan-Meier analysis of LADC patients in TCGA on the expression of G64. Kaplan-Meier survival analysis combined with the log-rank test was performed, and Cox regression analysis was used to investigate whether the survival duration of the two different groups, i.e., the G64 up-regulated group and the G64 down-regulated group, were significantly different.

Using the LADC patient survival data and the TCGA expression data, Kaplan-Meier analysis was performed to investigate how the up-/down-regulation of G64 is related to patient survival. The G64 up-regulated patient group exhibited significantly worse survival rates than the G64 down-regulated patient group based on Cox regression analysis (Fig 8) (p < 0.05). Each G64 gene was investigated to determine whether its up-regulation or down-regulation correlates with patient survival. We found that the up-regulation of 18 genes among the G64 genes were significantly associated with poor patient prognosis (S9 Fig and S4 Table). The same finding, i.e., the association of poor prognosis with G64 up-regulation, was confirmed in the 88 Korean lung cancer samples (data not shown).

## Discussion

The single-cell genomics approach has received considerable attention lately because of the efforts to deeply understand the genetic heterogeneity of cancers at a single-cell level. Along these lines, we identified a potentially important tumor subpopulation at a phenotypic expression level using transcriptomic data for single cells from PDXs.

The mouse xenograft and cell culture models have broadly been used for the expansion of human tumors for further molecular characterization and drug screening [53,54]. However, the xenograft-based single-cell approach may essentially produce unwanted gene expression artifacts based on transcriptomic analysis of single tumor cells. Therefore, we designed and performed complex and extensive data cleaning procedures at the beginning of our study to obtain intrinsic tumor-associated gene expression data in single cells. The essence of the cleaning strategy was to exclude DEGs that were altered by the xenografting procedure, and these DEGs were identified by comparing the gene expression profiles between the primary tumor and the xenograft tumor. Additionally, DEGs altered by the cell-culture procedure were identified by comparing the gene expression profiles between the xenograft tumor and the cultured cells and between the primary tumor and the cultured cells.

Our gene selection strategy after extensive cleaning was devised to overcome the noisy gene expression associated with single-cell transcriptomic data. Single-cell transcriptomic analysis of mixed tumor populations may reveal clinically important subpopulations at a phenotypic level. However, this strategy has limitations associated with uneven or incomplete amplification of the scarce starting material; thus, individual gene expression can be extremely noisy [48–50]. Our strategy to overcome this problem was to select groups of genes that were highly correlatively expressed across single cells. This method has long been used to detect genes acting as a ‘module’ in gene-gene interaction networks in microarray data [41,55,56]. In brief, we first identified 20 core seed genes displaying a Pearson’s correlation coefficient > = 0.9 and expanded this group by applying a loose threshold (0.75), ultimately identifying a set of 64 genes termed G64 (Fig 2).

The 34 single cells were clearly classified into subgroups based on G64 up-/down-regulation (Fig 3). Interestingly, the expression of G64 separated LADC patient groups, as well (Figs 4 and 5). This finding suggests that a different mixture of heterogeneous cell populations may result in inter-tumoral differences obtained using the G64 module, implying that dissecting intra-tumoral heterogeneity could lead to a better understanding of inter-tumoral heterogeneity. Forty of the G64 genes were actually cell-cycle genes (Fig 6 and S1 Table), and they were assigned to all cell-cycle stages. This study did not resolve how the function of cell cycle regulation is related to these distinct intra- and inter-tumor subgroups. However, it is apparent that their up-regulation and down-regulation may serve as a good prognostic indicator of LADC patient survival. These findings suggest that studies at the single-cell level could ultimately lead to the development of effective cancer treatment strategies avoiding drug resistance.

## Supporting Information

S1 Fig Schematic illustrating how gene expression fluctuated according to the experimental procedures. (A) Schematic showing the types of experimental procedures that might contribute to the generation of DEGs when a normal tissue has become a bulk tumor (i.e., a collection of single cells) according to the procedures used to obtain single cells described by Kim et al. [28]. Please refer to the Materials and Methods section for more detailed information. (B) Illustration showing a total of 9,455 genes located in the area filled with oblique lines after cleaning genes according to the procedure shown in (A).
(PDF)

S2 Fig Schematic showing two different types of genes displaying simple expression patterns. (A) Simple expression pattern of a gene, i.e., a gene expressed in one or two single cells and displaying consistently negligible FPKM values among the remaining cells. (B) Expression pattern of a housekeeping gene (i.e., a gene expressed or fluctuating across all 34 single cells from -3 to 3 of the log2 fold-change).
(PDF)

S3 Fig Schematic illustrating tumor tissue types according to the experimental preparation steps and the correlations in gene expression between these tumor types. (A) Box plot analyses of the Pearson’s correlation coefficients between the groups. The sizes of the correlation coefficients are indicated by short colored bars, and each color exactly corresponds to the results shown in (B). (B) Illustration of the correlation in gene expression according to the tumor tissue types obtained from each single-cell preparation step. The numbers inside the colored arrows are Pearson’s correlation coefficients (see the Materials and Methods section).
(PDF)

S4 Fig Comparison of the correlations in expression between pT and bulkT before and after cleaning. (A) Expression correlation before cleaning between pT and bulkT. (B) Expression correlation after cleaning between pT and bulkT. The red lines in each box are regression lines. The ‘*r*’ inside each box is Pearson’s correlation coefficient.
(PDF)

S5 Fig Comparison of the correlation coefficients within gene groups identified using different thresholds. Using a higher correlation coefficient threshold essentially provides fewer genes in the groups. However, following the use of the higher correlation coefficient, we selected the threshold r > 0.75 to identify correlative modules considering a balance between the correlation strength and the number of genes within a module.
(PDF)

S6 Fig Heat map combined with hierarchical clustering analysis of G64 expression in 501 LUSC samples in TCGA. The same heat map analysis described in the legend of Fig 3A was performed on RNA-seq data derived from the 501 LUSC samples downloaded from TCGA.
(PDF)

S7 Fig Proportion of genes among G64 containing a corresponding upstream TFBS. Considering that the coordinately expressed genes were likely to be regulated by common TFs, we investigated whether TFBSs were located within 5 kb upstream of G64 genes using ChipBase (see the Materials and Methods section). A total of 19 TFs were predicted to contain a corresponding upstream TFBS in at least 32 genes among the G64 module (i.e., > = 50%).
(PDF)

S8 Fig Box plot of correlation coefficients of gene expression between E2F1 and G64 based on TCGA data. The same analysis as that shown in Fig 6 was performed using 488 TCGA samples. Pearson’s correlation coefficient was estimated for the relationship between the expression of each of the TFs that were predicted to contain a TFBS at putative promoter regions of G64 and the expression of each gene of the G64 in the 488 TCGA samples. E2F1 displayed the highest correlation coefficient.
(PDF)

S9 Fig Kaplan-Meier analysis of the expression of each selected gene among LADC patients in TCGA. Kaplan-Meier survival analysis of the 18 selected genes is shown. The up-/down-regulation of these 18 genes exhibited a significant survival difference at p <0.05 based on Cox regression analysis. The up-regulation of these 18 genes was consistently related to poor prognosis.
(PDF)

S1 Table List of 20 seed genes and the genes in G64. (DOCX)

S2 Table Chi-square test and univariate logistic regression analysis of the relationship between G64 expression and clinical factors. (DOCX)

S3 Table GO classification of G64 genes using DAVID. (DOCX)

S4 Table Risk ratio estimated by Cox hazard regression analysis. (DOCX)
