# Supplementary figures and images for: Identification of Distinct Tumor Subpopulations in Lung Adenocarcinoma via Single-Cell RNA-seq
Source: PLoS One. 2015 Aug 25;10(8):e0135817. doi: 10.1371/journal.pone.0135817 (PMC4549254; doi:10.1371/journal.pone.0135817)

**A**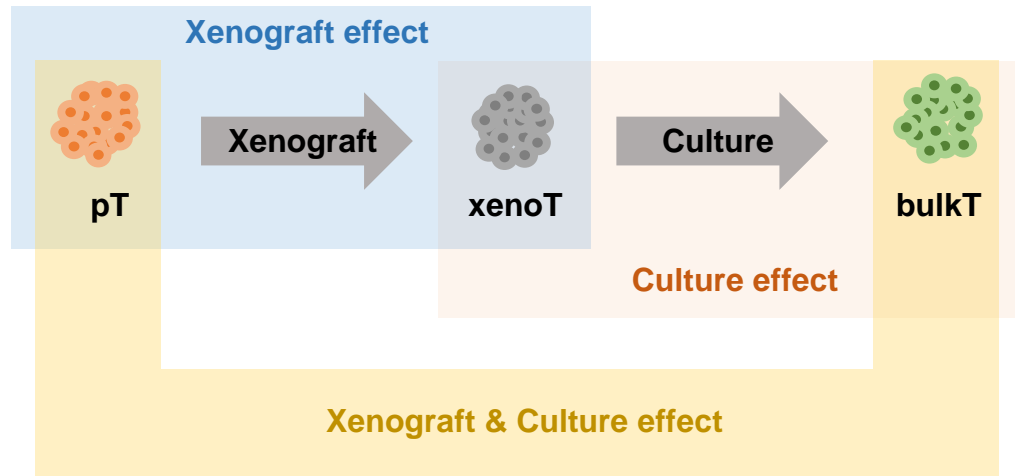**B**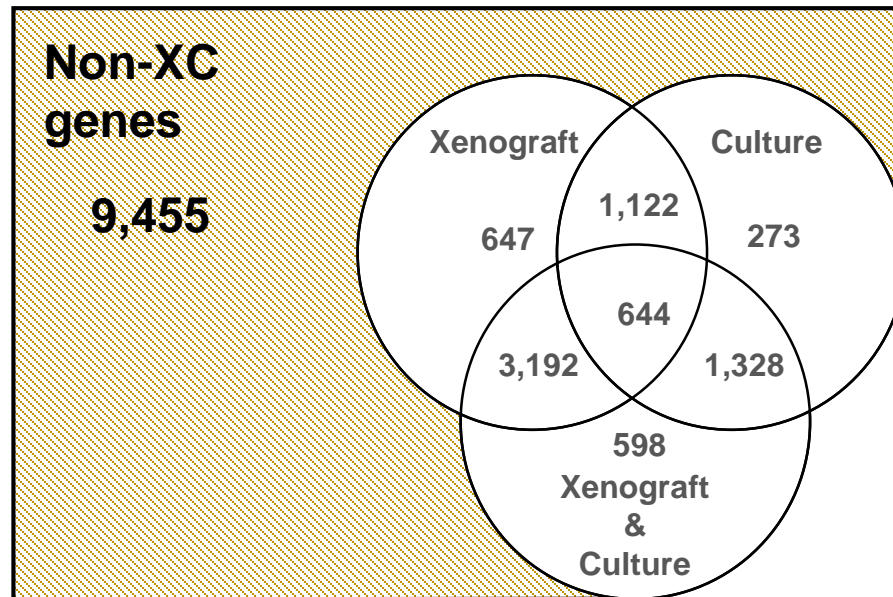

Supplement: S1 Fig — (A) Schematic showing the types of experimental procedures that might contribute to the generation of DEGs when a normal tissue has become a bulk tumor (i.e., a collection of single cells) according to the procedures used to obtain single cells described by Kim et al. [28]. Please refer to the Materials and Methods section for more detailed information. (B) Illustration showing a total of 9,455 genes located in the area filled with oblique lines after cleaning genes according to the procedure shown in (A). (PDF) [file pone.0135817.s001.pdf]

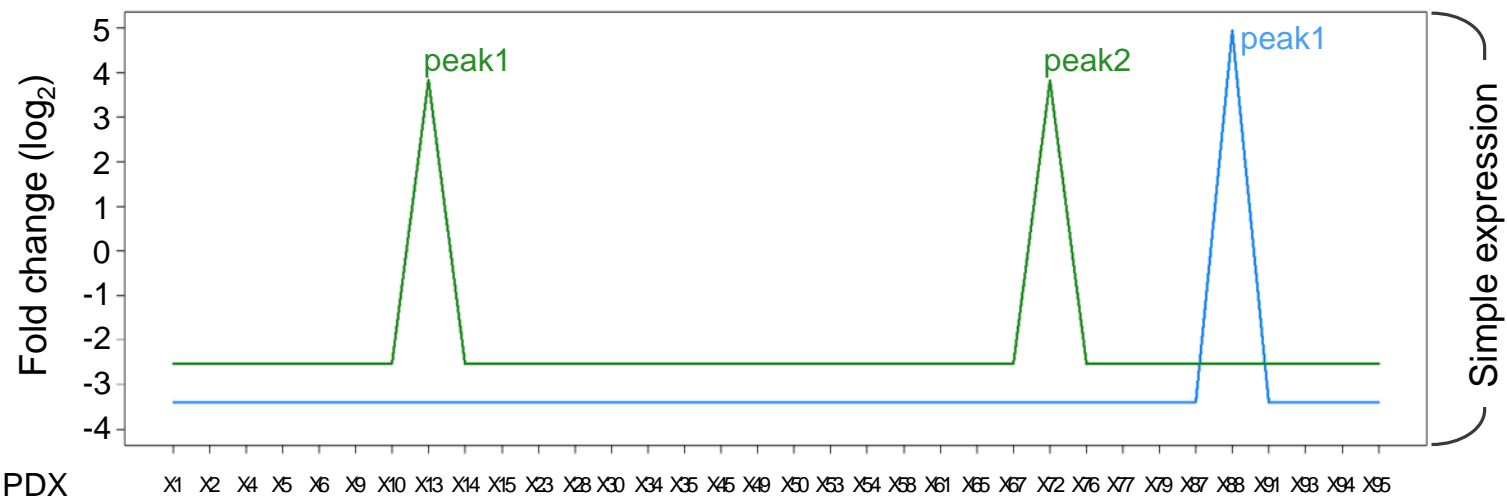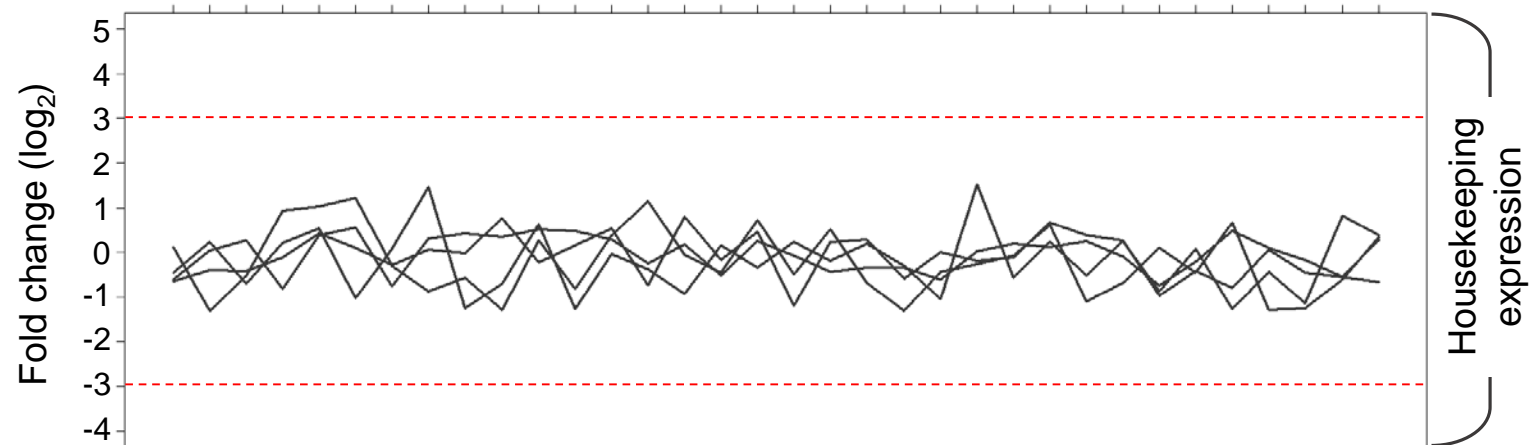

Supplement: S2 Fig — (A) Simple expression pattern of a gene, i.e., a gene expressed in one or two single cells and displaying consistently negligible FPKM values among the remaining cells. (B) Expression pattern of a housekeeping gene (i.e., a gene expressed or fluctuating across all 34 single cells from -3 to 3 of the log2 fold-change). (PDF) [file pone.0135817.s002.pdf]

**A**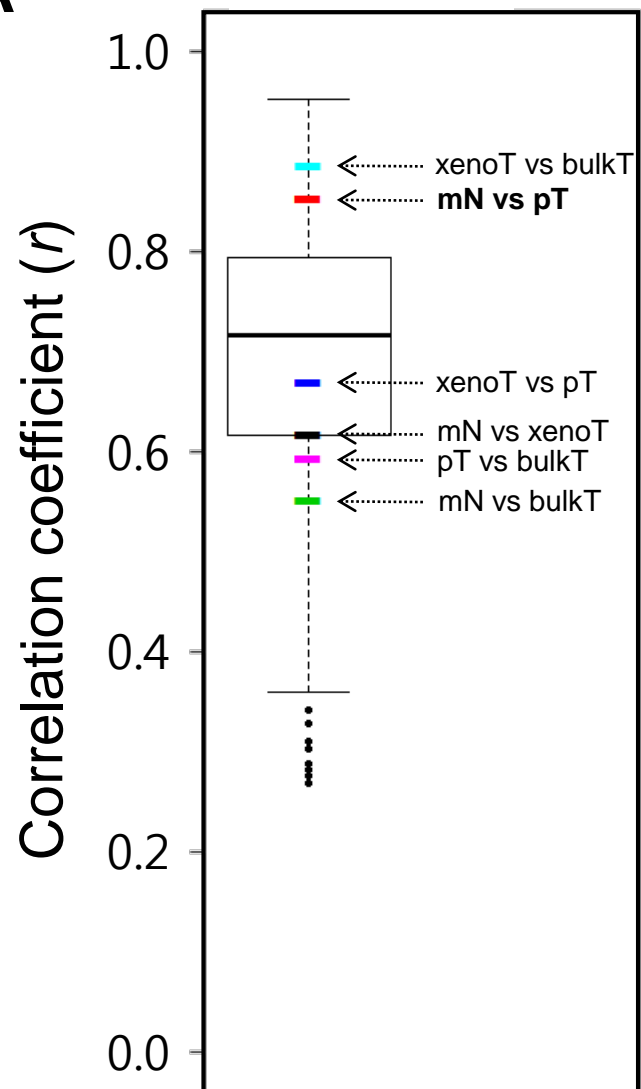**B**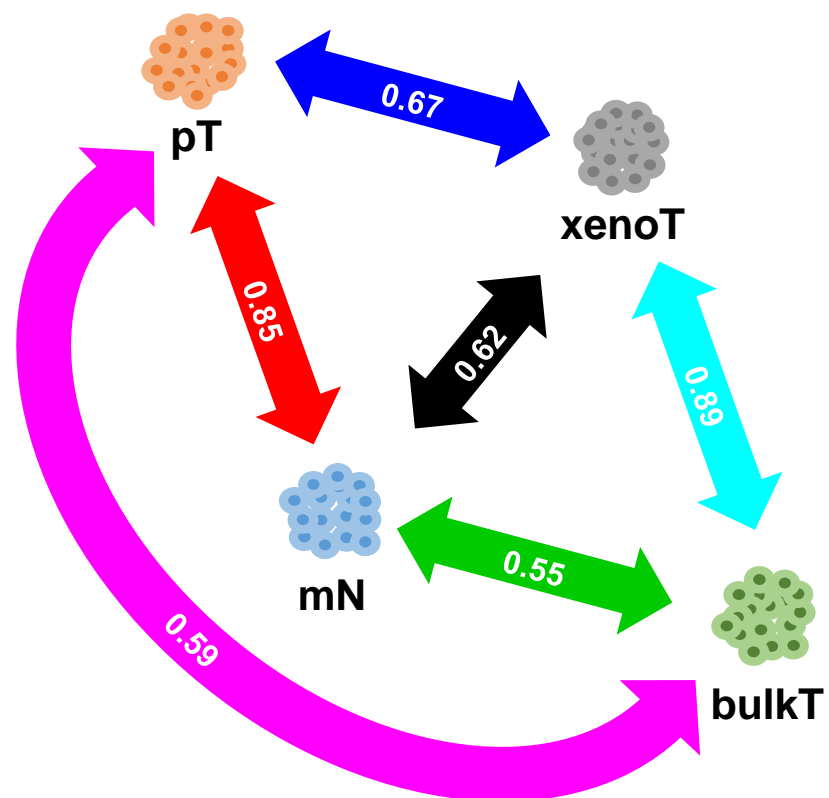

Supplement: S3 Fig — (A) Box plot analyses of the Pearson’s correlation coefficients between the groups. The sizes of the correlation coefficients are indicated by short colored bars, and each color exactly corresponds to the results shown in (B). (B) Illustration of the correlation in gene expression according to the tumor tissue types obtained from each single-cell preparation step. The numbers inside the colored arrows are Pearson’s correlation coefficients (see the Materials and Methods section). (PDF) [file pone.0135817.s003.pdf]

**A**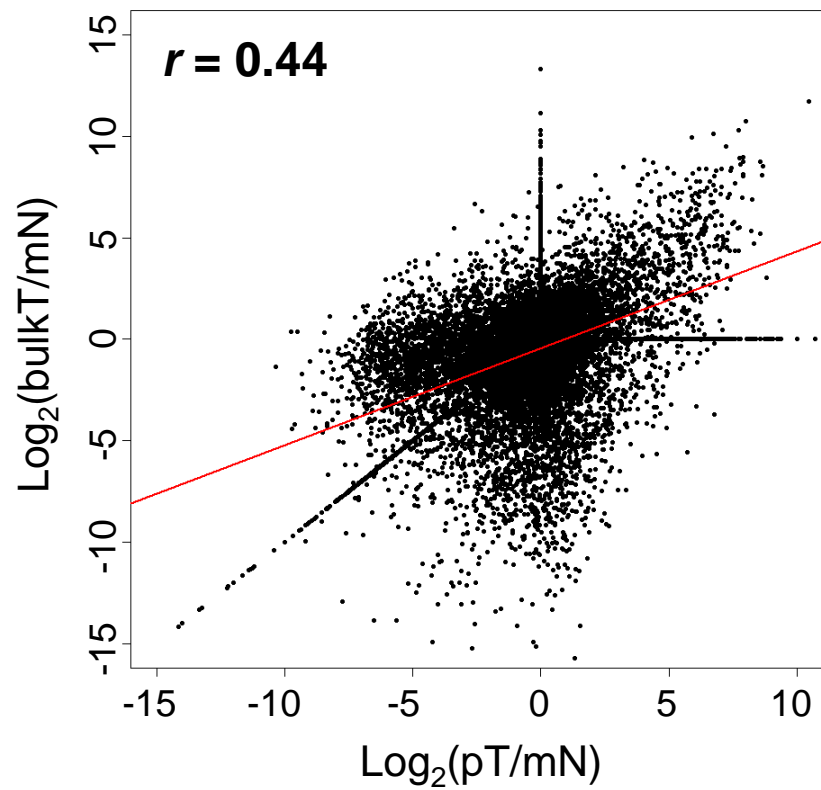**B**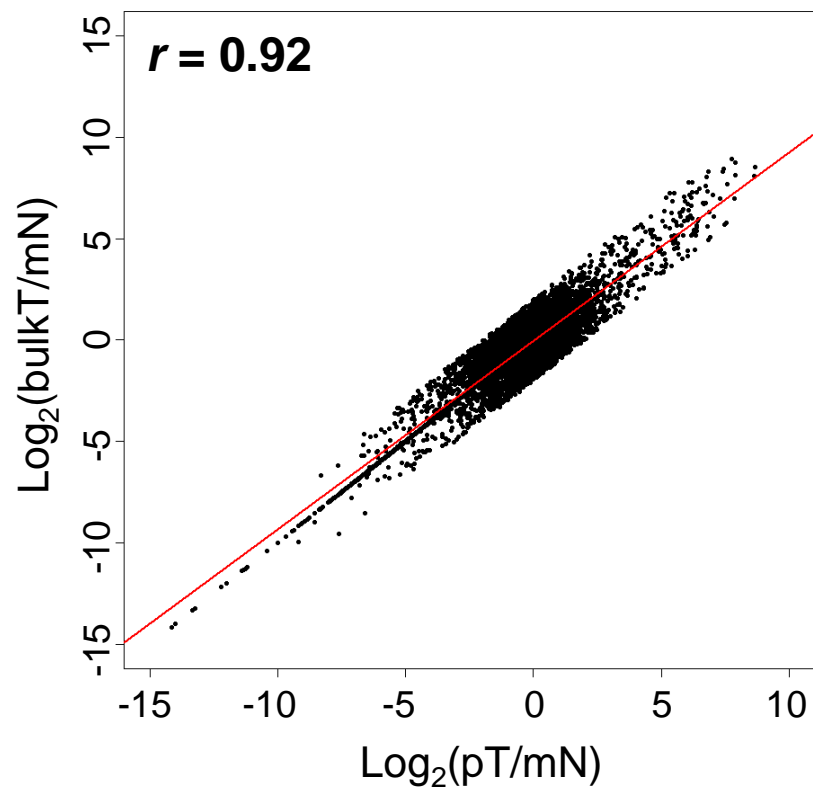

Supplement: S4 Fig — (A) Expression correlation before cleaning between pT and bulkT. (B) Expression correlation after cleaning between pT and bulkT. The red lines in each box are regression lines. The ‘r’ inside each box is Pearson’s correlation coefficient. (PDF) [file pone.0135817.s004.pdf]

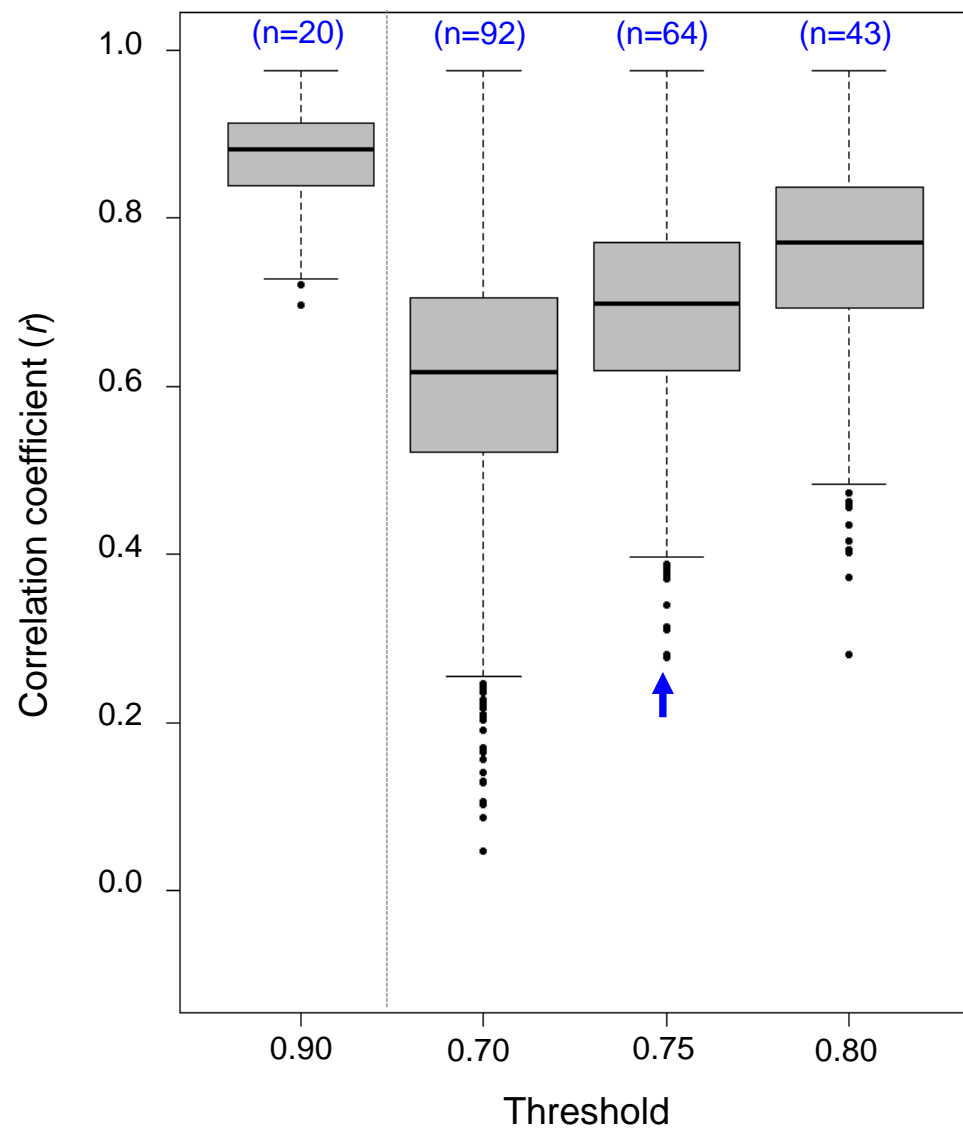

Supplement: S5 Fig — Using a higher correlation coefficient threshold essentially provides fewer genes in the groups. However, following the use of the higher correlation coefficient, we selected the threshold r > 0.75 to identify correlative modules considering a balance between the correlation strength and the number of genes within a module. (PDF) [file pone.0135817.s005.pdf]

Foldchange(Log<sub>2</sub>)

-4 -2 0 2 4

G64

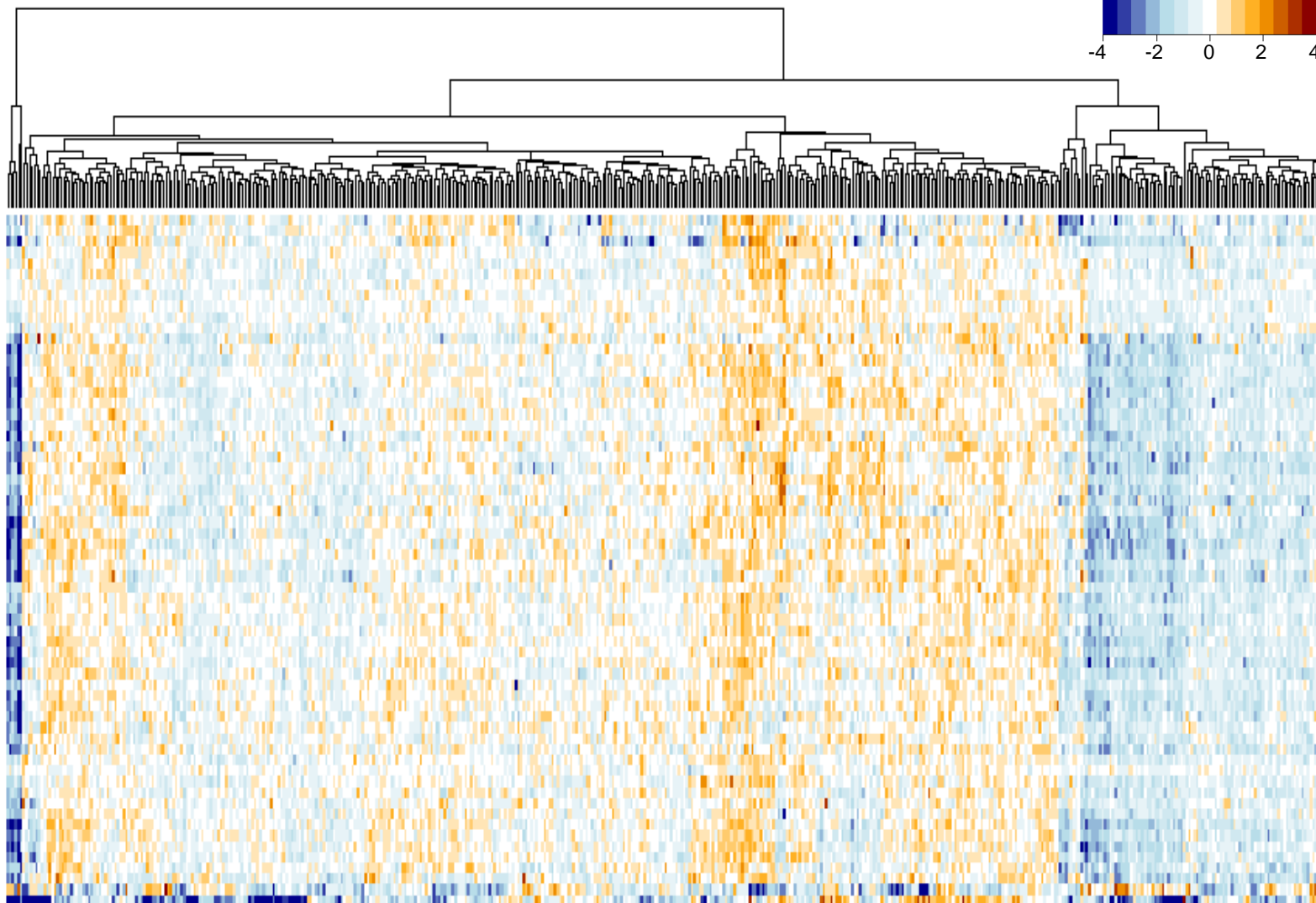

TCGA LUSC samples (n = 501)

Supplement: S6 Fig — The same heat map analysis described in the legend of Fig 3A was performed on RNA-seq data derived from the 501 LUSC samples downloaded from TCGA. (PDF) [file pone.0135817.s006.pdf]

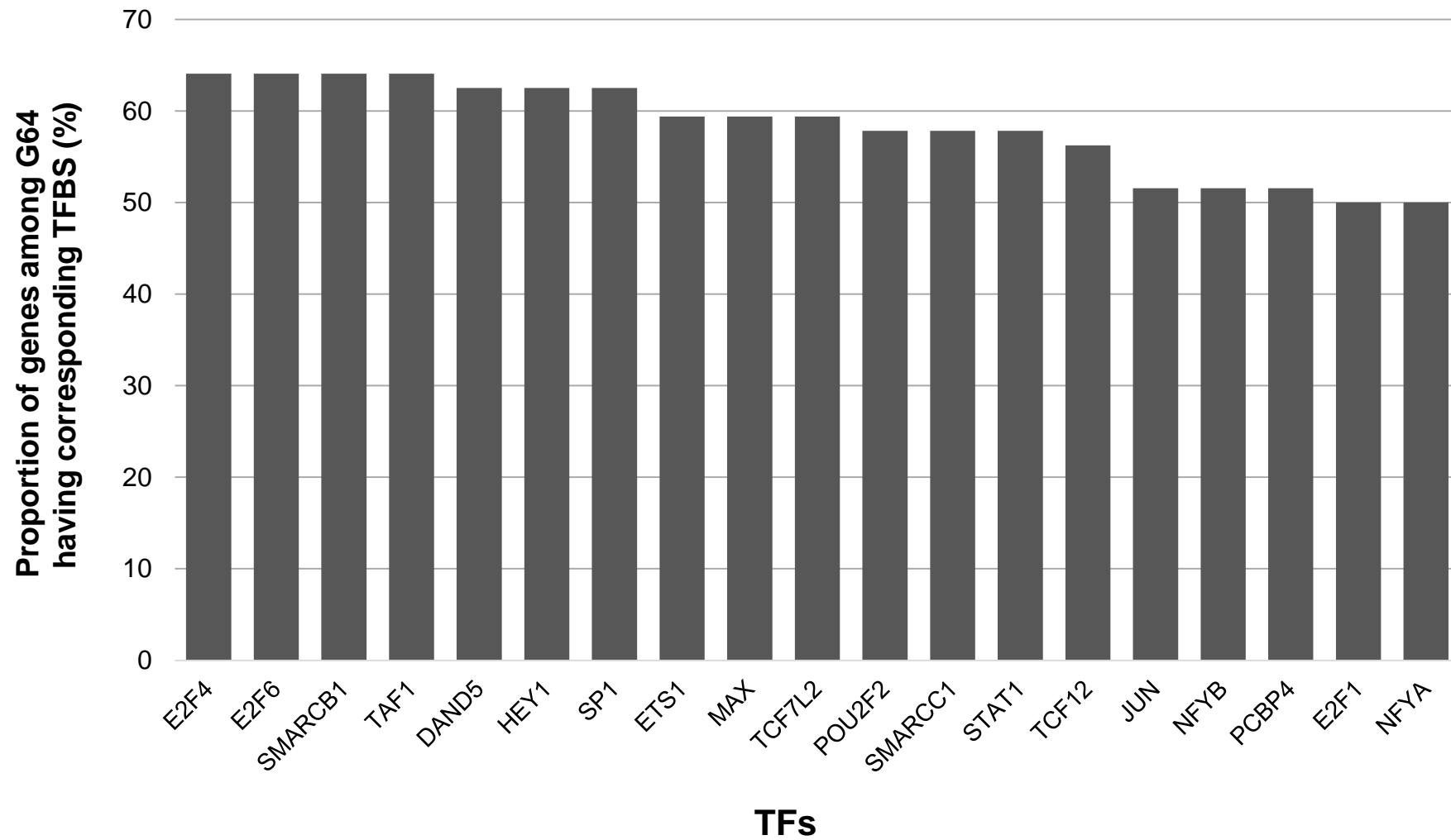

Supplement: S7 Fig — Considering that the coordinately expressed genes were likely to be regulated by common TFs, we investigated whether TFBSs were located within 5 kb upstream of G64 genes using ChipBase (see the Materials and Methods section). A total of 19 TFs were predicted to contain a corresponding upstream TFBS in at least 32 genes among the G64 module (i.e., > = 50%). (PDF) [file pone.0135817.s007.pdf]

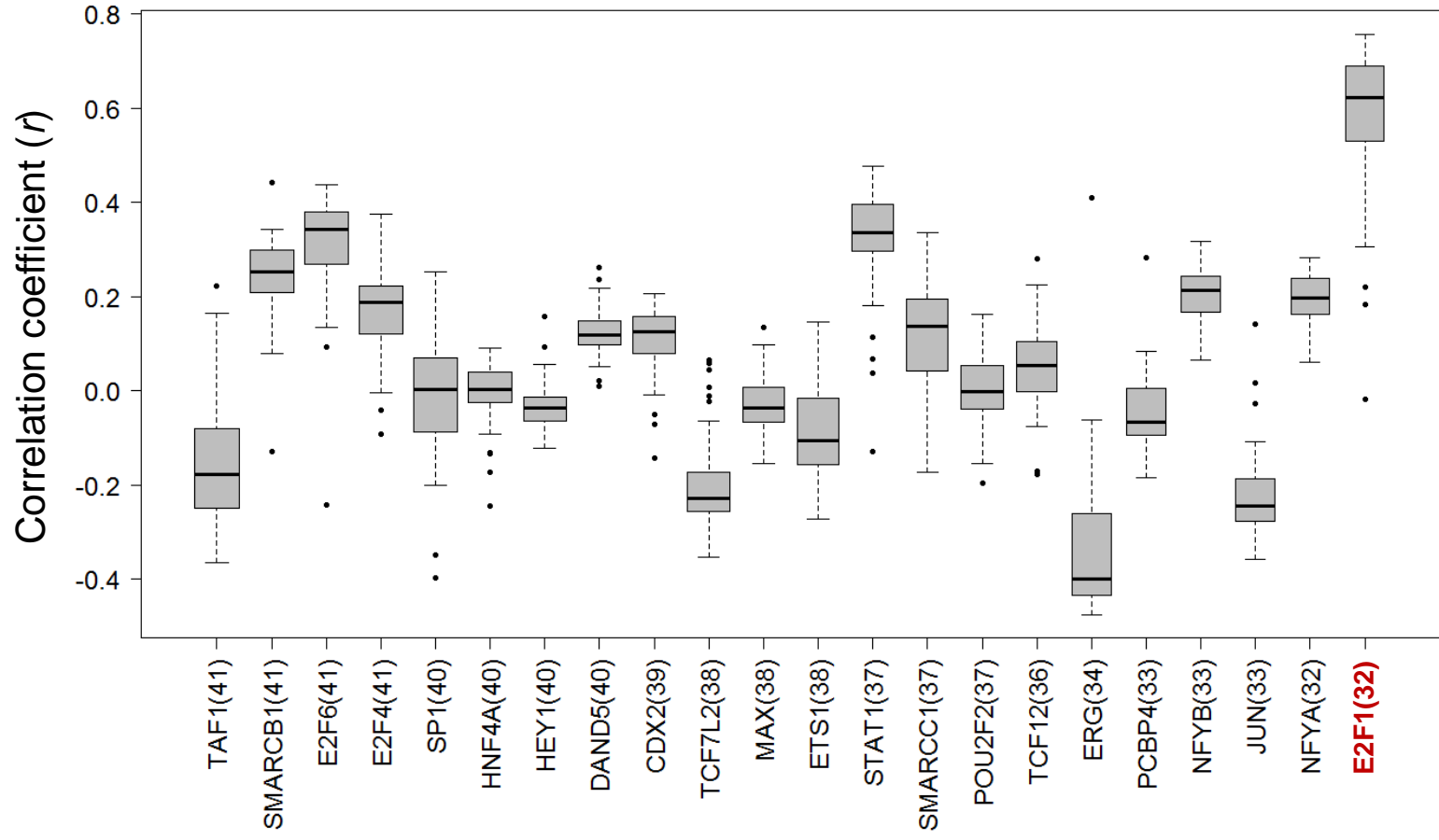

Supplement: S8 Fig — The same analysis as that shown in Fig 6 was performed using 488 TCGA samples. Pearson’s correlation coefficient was estimated for the relationship between the expression of each of the TFs that were predicted to contain a TFBS at putative promoter regions of G64 and the expression of each gene of the G64 in the 488 TCGA samples. E2F1 displayed the highest correlation coefficient. (PDF) [file pone.0135817.s008.pdf]

Survival probability

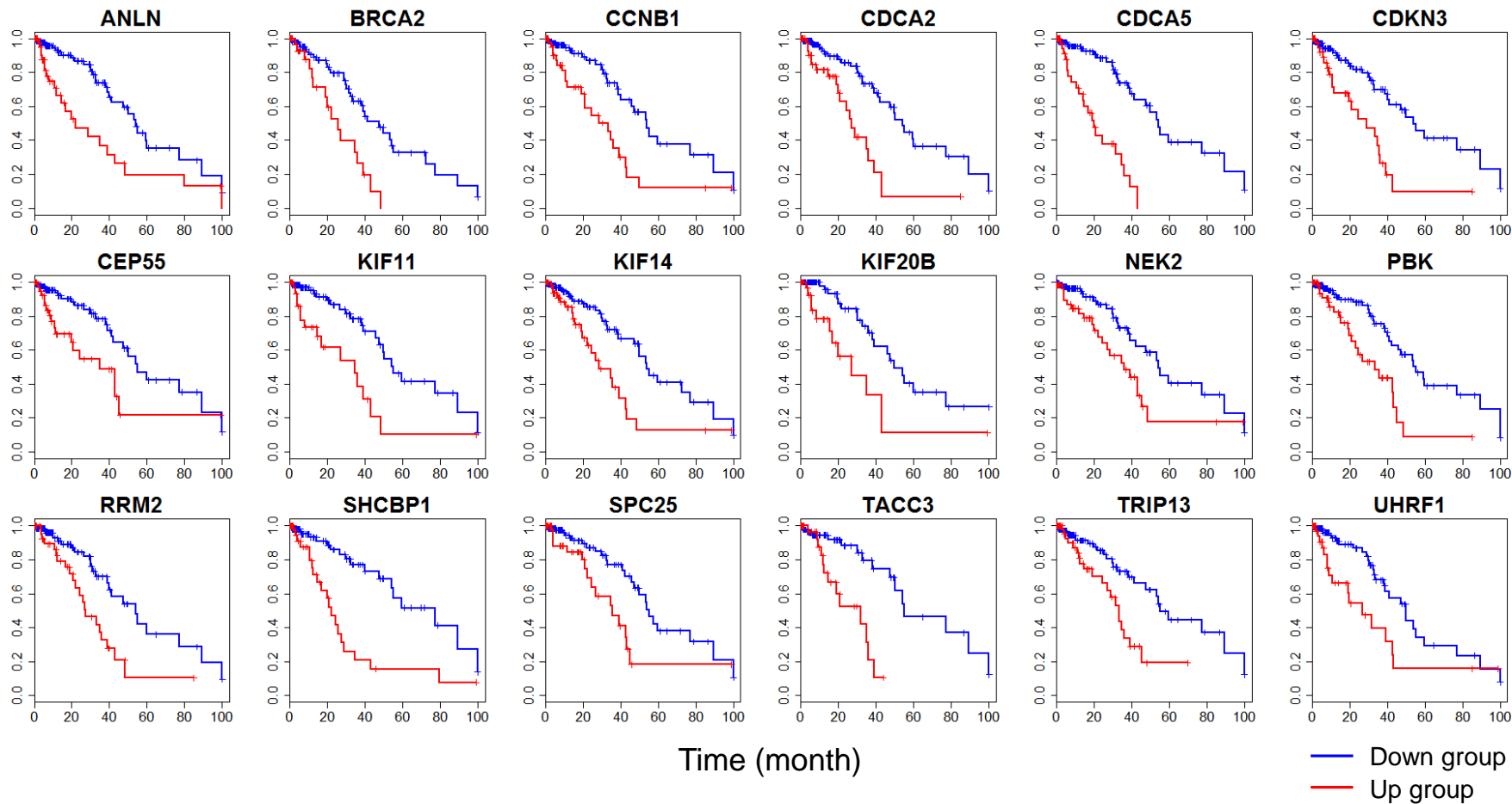

Supplement: S9 Fig — Kaplan-Meier survival analysis of the 18 selected genes is shown. The up-/down-regulation of these 18 genes exhibited a significant survival difference at p <0.05 based on Cox regression analysis. The up-regulation of these 18 genes was consistently related to poor prognosis. (PDF) [file pone.0135817.s009.pdf]
