# Supplementary material for: Identification of Distinct Tumor Subpopulations in Lung Adenocarcinoma via Single-Cell RNA-seq
Source: PLoS One. 2015 Aug 25;10(8):e0135817. doi: 10.1371/journal.pone.0135817 (PMC4549254; doi:10.1371/journal.pone.0135817)
Supplement: S1 Table — (DOCX) [file pone.0135817.s010.docx]

**S1 Table. List of 20 seed genes and the genes in G64**

Gene Symbol Gene Description

**ANLN** anillin, actin binding protein

**CDCA2** cell division cycle associated 2

**CDCA8** cell division cycle associated 8

**CENPK** centromere protein K

**CEP55** centrosomal protein 55kDa

**DLGAP5** discs, large (Drosophila) homolog-associated protein 5

**ESPL1**  extra spindle pole bodies homolog 1 (S. cerevisiae)

**HIST1H3B** histone cluster 1, H3b

**KIF14**  kinesin family member 14

**KIF11** kinesin family member 11

**KIF20B** kinesin family member 20B

**MCM10** minichromosome maintenance complex component 10

**MLF1IP** MLF1-Interacting Protein

**NDC80**  NDC80 kinetochore complex component

**NUSAP1** nucleolar and spindle associated protein 1

**PBK** PDZ binding kinase

**RRM2**  ribonucleotide reductase M2

**SHCBP1** SHC SH2-domain binding protein 1

**TROAP** trophinin associated protein

**ZWINT** ZW10 interacting kinetochore protein

ASF1B anti-silencing function 1B histone chaperone

ATAD2 ATPase family, AAA domain containing 2

AURKB aurora kinase B

BARD1 BRCA1 associated RING domain 1

BRCA2 breast cancer 2, early onset

C9orf100 Rho Guanine Nucleotide Exchange Factor (GEF) 39

CAV1 caveolin 1, caveolae protein, 22kDa

CCNB1 cyclin B1

CDCA5 cell division cycle associated 5

CDKN3 cyclin-dependent kinase inhibitor 3

CENPE centromere protein E, 312kDa

CENPM centromere protein M

CENPN centromere protein N

CENPW centromere protein W

CKS1B CDC28 protein kinase regulatory subunit 1B

DDX39A DEAD (Asp-Glu-Ala-Asp) box polypeptide 39A

FAM111B family with sequence similarity 111, member B

GAS2L3 growth arrest-specific 2 like 3

GEN1 GEN1 Holliday junction 5' flap endonuclease

GMNN geminin, DNA replication inhibitor

HMGB1 high mobility group box 1

HMGN2 high mobility group nucleosomal binding domain 2

ITGB3BP integrin beta 3 binding protein (beta3-endonexin)

KIAA1524 KIAA1524

KIF22 kinesin family member 22

MCM3 minichromosome maintenance complex component 3

MCM4 minichromosome maintenance complex component 4

MTBP MDM2 binding protein

NCAPG2 non-SMC condensin II complex, subunit G2

NCAPH non-SMC condensin I complex, subunit H

NEK2 NIMA-related kinase 2

NRM nurim (nuclear envelope membrane protein)

PARPBP PARP1 binding protein

PTMA prothymosin, alpha

RAD51AP1 RAD51 associated protein 1

RAD54B RAD54 homolog B (S. cerevisiae)

RFC2 replication factor C (activator 1) 2, 40kDa

SPC25 SPC25, NDC80 kinetochore complex component

TACC3 transforming, acidic coiled-coil containing protein 3

TRIP13 thyroid hormone receptor interactor 13

TUBA1B tubulin, alpha 1b

UHRF1 ubiquitin-like with PHD and ring finger domains 1

USP1 ubiquitin specific peptidase 1

VRK1 vaccinia related kinase 1

Seed genes are indicated by bold face.
