# Supplementary material for: Identification of Distinct Tumor Subpopulations in Lung Adenocarcinoma via Single-Cell RNA-seq
Source: PLoS One. 2015 Aug 25;10(8):e0135817. doi: 10.1371/journal.pone.0135817 (PMC4549254; doi:10.1371/journal.pone.0135817)
Supplement: S2 Table — (DOCX) [file pone.0135817.s011.docx]

**S2 Table. Chi-square test and univariate logistic regression analysis of the relationship between G64 expression and clinical factors**

| Factor | | Group | | *P* | O.R. | 95% C.I. |
| --- | --- | --- | --- | --- | --- | --- |
|  |  | Down(228) | Up(234) |  |  |  |
| Gender | female | 140(55.6) | 112(44.4) | 0.003 | 1.731 | 1.177~2.553 |
|  | male | 88(41.9) | 122(58.1) |  |  |  |
| Age of initial  pathologic diagnosis | >=65 | 140(55.8) | 111(44.2) | 0.005 | 1.726 | 1.162~2.573 |
|  | <65 | 81(42.2) | 111(57.8) |  |  |  |
| Stage | 1~2 | 190(53.1) | 168(46.9) | 0.002 | 2.014 | 1.255~3.270 |
|  | 3~4 | 37(35.9) | 66(64.1) |  |  |  |
| Tobacco | No | 43(59.7) | 29(40.3) | 0.043 | 1.690 | 0.985~2.935 |
|  | Yes | 177(46.7) | 202(53.3) |  |  |  |
| Tobacco year | <35 | 58(54.7) | 48(45.3) | 0.052 | 1.648 | 0.964~2.831 |
|  | >=35 | 60(42.3) | 82(57.7) |  |  |  |
| Tobacco reformed  year | >=15 | 79(68.7) | 36(31.3) | 0.000 | 3.106 | 1.824~5.362 |
|  | <15 | 64(41.3) | 91(58.7) |  |  |  |
| Tobacco pack-  years | <42 | 98(52.1) | 90(47.9) | 0.008 | 1.850 | 1.141~3.018 |
|  | >=42 | 47(37.0) | 80(63.0) |  |  |  |
| Vital | Survival | 188(54.2) | 159(45.8) | 0.000 | 2.213 | 1.401~3.533 |
|  | Death | 40(34.8) | 75(65.2) |  |  |  |

O.R.: odds ratio; C.I.: confidence interval; *P*: p value
