# Supplementary material for: Identification of Distinct Tumor Subpopulations in Lung Adenocarcinoma via Single-Cell RNA-seq
Source: PLoS One. 2015 Aug 25;10(8):e0135817. doi: 10.1371/journal.pone.0135817 (PMC4549254; doi:10.1371/journal.pone.0135817)
Supplement: S3 Table — (DOCX) [file pone.0135817.s012.docx]

**S3 Table. GO classification of G64 genes using DAVID**

| Gene Ontology | Count | Genes | P value |
| --- | --- | --- | --- |
| cell cycle | 33 | GAS2L3, CKS1B, KIF22, NEK2, ANLN, CEP55, AURKB, SPC25, CDCA8, NCAPH, NCAPG2,  CDCA2, MTBP, CDCA5, TRIP13, KIF11, GMNN, DLGAP5, BRCA2, NUSAP1, CENPE, NDC80,  ESPL1, PBK, CDKN3, MCM3, TACC3, CCNB1, UHRF1, ZWINT, KIF20B, RAD54B, BARD1 | 1.35E-23 |
| cell cycle phase | 27 | KIF22, NEK2, ANLN, CEP55, AURKB, SPC25, CDCA8, NCAPH, NCAPG2, CDCA2, MTBP,  CDCA5, TRIP13, KIF11, DLGAP5, BRCA2, NUSAP1, CENPE, NDC80, ESPL1, PBK, TACC3, CDKN3, CCNB1, ZWINT, KIF20B, RAD54B | 9.56E-23 |
| M phase | 25 | KIF22, NEK2, ANLN, CEP55, AURKB, SPC25, CDCA8, NCAPH, NCAPG2, CDCA2, CDCA5,  TRIP13, KIF11, DLGAP5, BRCA2, NUSAP1, CENPE, NDC80, ESPL1, PBK, TACC3, CCNB1,  ZWINT, KIF20B, RAD54B | 3.29E-22 |
| cell cycle process | 29 | GAS2L3, KIF22, NEK2, ANLN, CEP55, AURKB, SPC25, CDCA8, NCAPH, NCAPG2, CDCA2,  MTBP, CDCA5, TRIP13, KIF11, DLGAP5, BRCA2, NUSAP1, CENPE, NDC80, ESPL1, PBK,  CDKN3, TACC3, CCNB1, ZWINT, KIF20B, RAD54B, BARD1 | 4.83E-22 |
| mitosis | 21 | KIF22, KIF11, NEK2, DLGAP5, NUSAP1, ESPL1, NDC80, CENPE, ANLN, AURKB, PBK, CEP55, CCNB1, SPC25, NCAPH, CDCA8, NCAPG2, ZWINT, CDCA2, KIF20B, CDCA5 | 7.93E-20 |
| nuclear division | 21 | KIF22, KIF11, NEK2, DLGAP5, NUSAP1, ESPL1, NDC80, CENPE, ANLN, AURKB, PBK, CEP55, CCNB1, SPC25, NCAPH, CDCA8, NCAPG2, ZWINT, CDCA2, KIF20B, CDCA5 | 7.93E-20 |
| M phase of mitotic cell cycle | 21 | KIF22, KIF11, NEK2, DLGAP5, NUSAP1, ESPL1, NDC80, CENPE, ANLN, AURKB, PBK, CEP55, CCNB1, SPC25, NCAPH, CDCA8, NCAPG2, ZWINT, CDCA2, KIF20B, CDCA5 | 1.14E-19 |
| organelle fission | 21 | KIF22, KIF11, NEK2, DLGAP5, NUSAP1, ESPL1, NDC80, CENPE, ANLN, AURKB, PBK, CEP55, CCNB1, SPC25, NCAPH, CDCA8, NCAPG2, ZWINT, CDCA2, KIF20B, CDCA5 | 1.79E-19 |
