# Supplementary material for: Identification of Distinct Tumor Subpopulations in Lung Adenocarcinoma via Single-Cell RNA-seq
Source: PLoS One. 2015 Aug 25;10(8):e0135817. doi: 10.1371/journal.pone.0135817 (PMC4549254; doi:10.1371/journal.pone.0135817)
Supplement: S4 Table — (DOCX) [file pone.0135817.s013.docx]

**S4 Table. Risk ratio estimated by Cox hazard regression analysis**

| Gene | R.O. | 95% C.I. | *P* |
| --- | --- | --- | --- |
| ANLN | 2.56 | 1.47~4.44 | 0.001 |
| BRCA2 | 2.98 | 1.58~5.62 | 0.001 |
| CCNB1 | 2.67 | 1.50~4.75 | 0.001 |
| CDCA2 | 3.27 | 1.81~5.89 | 0.000 |
| CDCA5 | 5.70 | 3.04~10.69 | 0.000 |
| CDKN3 | 2.93 | 1.64~5.25 | 0.000 |
| CEP55 | 2.53 | 1.34~4.77 | 0.004 |
| KIF11 | 3.17 | 1.62~6.20 | 0.001 |
| KIF14 | 2.41 | 1.37~4.25 | 0.002 |
| KIF20B | 3.13 | 1.49~6.56 | 0.003 |
| NEK2 | 2.19 | 1.22~3.94 | 0.009 |
| PBK | 2.70 | 1.52~4.80 | 0.001 |
| RRM2 | 2.52 | 1.38~4.59 | 0.003 |
| SHCBP1 | 3.67 | 1.92~70 | 0.000 |
| SPC25 | 2.54 | 1.33~4.86 | 0.005 |
| TACC3 | 4.75 | 2.00~11.28 | 0.000 |
| TRIP13 | 2.50 | 1.35~4.63 | 0.003 |
| UHRF1 | 2.35 | 1.26~4.37 | 0.007 |
